# Bayesian hierarchical modeling: an introduction and reassessment

**DOI:** 10.3758/s13428-023-02204-3

**Published:** 2023-09-25

**Authors:** Myrthe Veenman, Angelika M. Stefan, Julia M. Haaf

**Affiliations:** 1 Leiden University, Wassenaarseweg 52, Leiden, Netherlands; 2 Universität der Bundeswehr München, Munich, Germany; 3 University of Amsterdam, Amsterdam, Netherlands

**Keywords:** Multilevel, Tutorial, *rstan*, *brms*, Bayes factor

## Abstract

With the recent development of easy-to-use tools for Bayesian analysis, psychologists have started to embrace Bayesian hierarchical modeling. Bayesian hierarchical models provide an intuitive account of inter- and intraindividual variability and are particularly suited for the evaluation of repeated-measures designs. Here, we provide guidance for model specification and interpretation in Bayesian hierarchical modeling and describe common pitfalls that can arise in the process of model fitting and evaluation. Our introduction gives particular emphasis to prior specification and prior sensitivity, as well as to the calculation of Bayes factors for model comparisons. We illustrate the use of state-of-the-art software programs Stan and *brms*. The result is an overview of best practices in Bayesian hierarchical modeling that we hope will aid psychologists in making the best use of Bayesian hierarchical modeling.

## Introduction

Imagine a typical scenario in the life of an experimental psychology graduate student: You have just collected a large data set, either online or in the lab. Each participant has spent one valuable hour completing many trials per experimental condition. Now it is time to analyze the data, and your supervisor suggests that you run a within-subjects analysis of variance (ANOVA). This procedure requires you to first aggregate the data by condition and participant. Doing so reduces your data set from 10,000 rows to just 400. Reducing the data by aggregation makes you a bit queasy. It feels like your efforts in data collection and the participants’ time are not valued. You know there needs to be a better way. And there is: hierarchical modeling.

Just as in the scenario above, experiments in psychology are often implemented in a repeated-measures design where participants respond to several items, stimuli, or conditions. From a data analysis perspective, such a design implies that observations are nested within participants. The statistical and the psychological literature agree that the optimal analysis accounting for such a data structure is hierarchical modeling (e.g., Efron & Morris, 1977; Lee, 2011; Rouder & Lu, 2005). And yet it is still not the norm to apply hierarchical modeling to data from psychological experiments. One reason for this is that hierarchical modeling is more difficult than familiar procedures such as ANOVA.

With the recent developments in software, hierarchical modeling has become increasingly accessible. In addition, many researchers have argued that hierarchical modeling is easier and the interpretation is more intuitive when it is done in a Bayesian statistical framework (e.g., Lynch, 2007; Rouder et al., 2013; Rouder & Lu, 2005). However, there are challenges to the Bayesian way of hierarchical modeling (Rouder & Lu, 2005). These challenges, such as the choice of priors, the need for model comparisons of highly complex models, and programming skills required to use state-of-the-art software solutions, can make researchers hesitant to use this approach. We believe that existing introductions to Bayesian hierarchical modeling do not adequately address these challenges. For example, they use outdated software (Rouder et al., 2013), do not cover model comparison (Rouder & Lu, 2005), or do not discuss prior specification (Shiffrin et al., 2008). This paper aims to make Bayesian hierarchical modeling available to a wider public by providing:

- a comparison of and guidance for the use of two software packages,
- guidance on prior specification, and
- practical instructions for Bayes factor model comparison.

Our tutorial is directed at researchers who have a basic conceptual understanding of Bayesian inference and parameter estimation and are planning to perform more complex Bayesian analyses of psychological phenomena. We hope to achieve a balance of detail and overview—while we want to provide guidance for a full Bayesian workflow, similar to Gelman et al. (2020) and Schad et al. (2021, 2022), we don’t shy away from in-depth explanations for the most curious scientists. To ensure that a broad audience is able to follow the tutorial, we limit and carefully explain equations. However, rudimentary knowledge of calculus is advantageous to fully understand the Bayesian analyses.

The application of Bayesian hierarchical analysis will be illustrated using a digit classification task collected by Rouder and Lu (2005). In an experimental task, participants repeatedly indicated whether a target digit (i.e., 2, 3, 4, 6, 7, or 8) was larger or smaller than 5. The participants in the task performed multiple trials per condition (i.e., digit). Therefore, observations are nested in participants. Our analyses focus on two effects that can potentially occur in this digit classification task: the digit effect and the side effect. According to the symbolic distance hypothesis (Moyer & Landauer, 1967), processing of numbers is an analog process. If numbers are closer on the number line, we may confuse them more, and comparing them is more difficult. In line with this hypothesis, the digit effect postulates that response times (RTs) are slower when digits are closer to 5. Additionally, we may hypothesize that RTs are affected by whether the target digit is smaller or larger than 5, yet it is unclear which side of 5 would lead to faster or slower RTs. The digit classification task is a standard task that usually would be analyzed in a 3 (digit, closest to 5, further away from 5, furthest away from 5) by 2 (side, greater or smaller than 5) within-subjects ANOVA. In this tutorial, we show how inferences can be improved using a hierarchical Bayesian model instead.

### Why multilevel modeling?

Before diving into hierarchical modeling, let us consider two alternative approaches: Analyzing aggregated data and analyzing the data separately for each participant. Analyses of aggregated data such as the ANOVA from the scenario above examine a general effect that is assumed to be consistent across subjects. For instance, when investigating whether there is an effect of digit on RT, the mean RT for each participant is computed for each digit-condition, and a repeated-measures ANOVA is conducted on these aggregated scores. Analyses of aggregated data cannot investigate whether a trend is consistent across participants. Therefore, when this option is applied and individual variability is ignored, the general trend can be an inaccurate representation of the true general trend (Haaf & Rouder, 2017; Rouder et al., 2013; van Doorn et al., 2021).

**Fig. 1:**
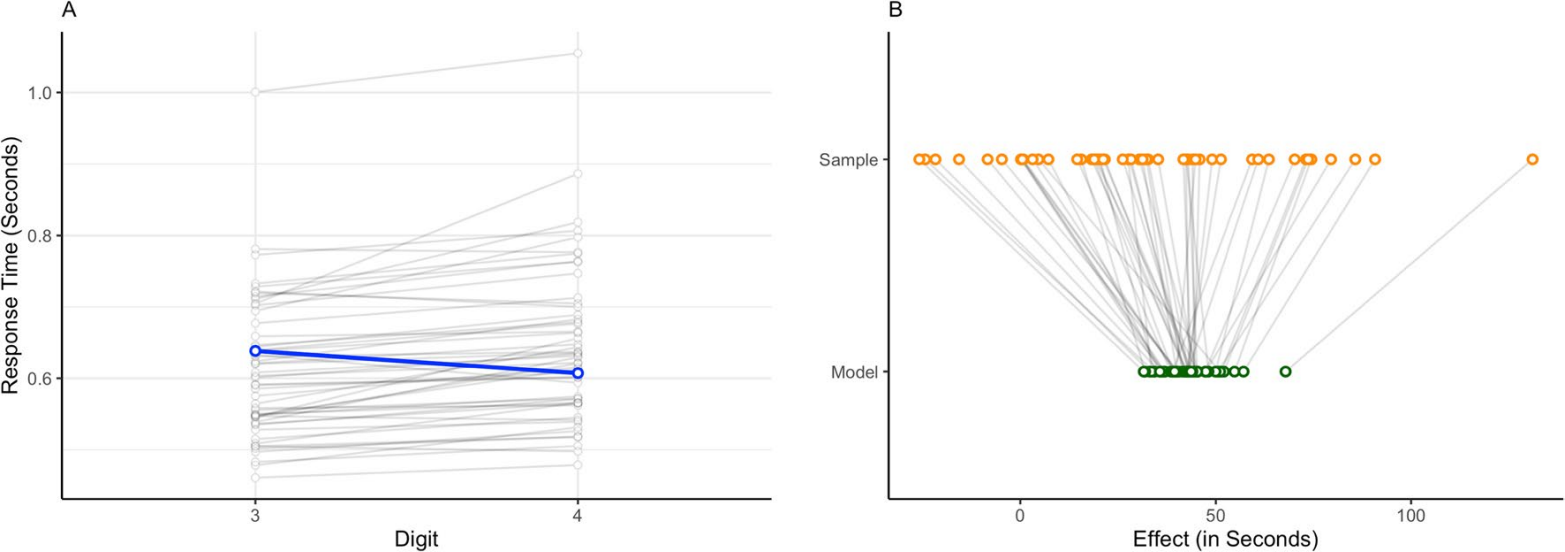
Panel **A** shows the average response times in seconds for two conditions of the digit classification task (i.e., digits 3 and 4). The gray lines represent the average inter-individual effects, and the blue line the aggregated effect over all individuals. Panel **B** shows how individual estimates shrink towards the general trend. Sample represents the observed effect per participant in seconds, while Model represents the estimated effects per participant

A pattern where the general trend does not reflect individual trends is shown in Panel A of Fig. 1. The slopes of the gray lines reflect individual trends, and some of them differ considerably from the general trend shown as the slope of the blue line. If every participant takes part in each condition, the variability among persons affects all conditions, thus inducing a correlation across conditions. For example, participants in a digit classification task repeatedly respond to target digits inducing a correlation between the responses to the target digits, such as between target digit 3 and target digit 4. Therefore, analyses on aggregated data do not appropriately account for differential experiment effects (van Doorn et al., 2021, 2023). As a result, the type I error rate of a test of the effect increases: there is a higher probability of concluding that there is an effect when there is no effect (Rouder & Lu, 2005).

A second alternative is to conduct an analysis on each participant’s data independently. For example, in the digit classification task, we would estimate one digit effect per person (cf. the gray lines in Fig. 1A), but no overall effect (cf. the blue line in Fig. 1A). Therefore, when analyzing participant data independently, it is hard to draw any general conclusions.

It is clear that neither of the alternative approaches yields satisfactory results by itself. Conceptually, a hierarchical analysis provides a compromise between the aggregated and independent approach by drawing benefits from each method. The motivation for hierarchical models, as well as their general mathematical structure, has been covered by several (tutorial) articles (e.g., Efron & Morris, 1977; Singmann & Kellen, 2019; West et al., 2022) as well as textbooks (e.g., Gelman & Hill, 2006; Hox et al., 2017; Nicenboim et al., 2023).

In hierarchical analyses, the general or overall effect is sometimes called the fixed effect, and the individual-specific deviations from this effect are in that case referred to as random effects (Rouder & Lu, 2005). We can also estimate individual effects, not as deviations from an overarching effect, but as effects for each individual, as obtained from a per-participant analysis. Throughout this tutorial, we will use the terms general effect, individual deviation, and individual effect.

If estimated for each participant independently, observed individual effects are quite variable because they are perturbed by sample noise. In hierarchical modeling, these individual effects are optimally and automatically corrected towards the general trend (Efron & Morris, 1977). This phenomenon, also called “shrinkage,” is illustrated in Panel B of Fig. 1. The rightmost observation in the figure in sample is quite large compared to the other observed effects. The estimated effect (green point) is drastically corrected towards the mean. Conversely, the general trend is only influenced slightly by individuals showing a highly divergent trend. By adding the individual variability to the model, we no longer assume independence between observations, but the overall effect in the model leaves open the possibility to generalize the effect. Therefore, hierarchical analysis can be considered a middle ground between an independent analysis and an analysis of aggregated data.

The Bayesian framework offers a more intuitive approach for hierarchical modeling (Lynch, 2007; Rouder & Lu, 2005). The flexibility of the Bayesian approach facilitates the implementation of complex hierarchical models (Rouder et al., 2013). It also allows for the inclusion of prior knowledge into the analysis, as well as the monitoring of evidence for specific hypotheses as the data accumulate (Wagenmakers et al., 2018). A complete discussion of the Bayesian approach is beyond the scope of this manuscript. We refer the interested reader to Etz and Vandekerckhove (2018) and Wagenmakers et al. (2018).

## Model specification

In the following section, we specify two models that can be used for our application example: a normal and a log-normal model.^1^ We are aware that many psychologists do not engage with equations on a daily basis. In this section, however, we try to accessibly summarize the key considerations of model specification. We encourage everyone to read on and not skip the section. Additionally, we provide a non-technical verbal summary.

Throughout the section, we assume that each observation *Y*_*ijk*_ is the response time from participant *i* in trial *k* of condition *j*. The condition *j* is the digit presented to the participant, and can take the values 2, 3, 4, 6, 7, and 8. We further assume that the digit side (*x*) is recorded using effect coding where a value of −0.5 indicates that the digit is smaller than 5, and a value of 0.5 indicates that the digit is larger than 5. Additionally, we assume that the digits presented in the trial are dummy-coded in the data using four variables (*u*, *v*, *w*, *z*). If digits 2 and 8 were presented, all four variables would be zero. The dummy variables represent the digits 3 (*u*), 4 (*v*), 6 (*w*), and 7 (*z*), and each take the value 1 if the respective digit was presented in the trial, and 0 if it was not (for more on contrast coding, we refer to Pinheiro & Bates, 2006; and Singmann & Kellen, 2019).

### Normal model

In the normal model, the observation *Y*_*ijk*_ is assumed to come from a normal distribution. The mean of this normal distribution is predicted by the person-specific intercept *γ*, the effect of the side (i.e., smaller or larger than 5, *β*) and the effect of the distance from 5 (i.e., symbolic distance effect, *δ*s). The variance is represented by *σ*^2^. This is captured in the following equation:

1 $${Y}_{ijk}\sim \mathrm{Normal}\left({\gamma}_i+{x}_j{\beta}_i+{u}_j{\delta}_{7i}+{v}_j{\delta}_{6i}+{w}_j{\delta}_{4i}+{z}_j{\delta}_{3i},{\sigma}^2\right),$$

where

$$\kern0.5em {\displaystyle \begin{array}{r}{x}_j=\left\{\begin{array}{ll}\frac{1}{2}& j<5\\ {}-\frac{1}{2}& j>5,\end{array}\right.\end{array}}\kern0.5em {\displaystyle \begin{array}{r}{u}_j=\left\{\begin{array}{ll}1& j=7\\ {}0& \mathrm{otherwise},\end{array}\right.\end{array}}\kern0.5em {\displaystyle \begin{array}{r}{v}_j=\left\{\begin{array}{ll}1& j=6\\ {}0& \mathrm{otherwise},\end{array}\right.\end{array}}$$

$${\displaystyle \begin{array}{rrr}& \begin{array}{r}{w}_j=\left\{\begin{array}{ll}1& j=4\\ {}0& \mathrm{otherwise},\end{array}\right.\end{array}& \begin{array}{r}{z}_j=\left\{\begin{array}{ll}1& j=3\\ {}0& \mathrm{otherwise}.\end{array}\right.\end{array}\end{array}}$$

The model equation enables us to compute the expected response time *Y*_*ij*_ for a participant *i* when presented with a specific probe. For example, if participant *i* = 1 is presented with digit *j* = 7, *x*_*j*_ takes the value $-\frac{1}{2}$ and *u*_*j*_ takes the value 1. The parameters *v*_*j*_, *w*_*j*_, and *z*_*j*_ take the value zero, such that the equation for the expected value reduces to: ${\gamma}_i+\frac{1}{2}{\beta}_i+{\delta}_{7,i}$. Let us assume that participant 1’s mean response time across all items is *γ*_1_ = 0.5, they respond generally faster to digits to the right of 5, as expressed by *β*_1_ = 0.2, and they respond slower to digit 7, given by *δ*_7, 1_ = 0.25. Then their expected response time for the digit 7 can be calculated by 0.5 − 0.5 × 0.2 + 1 × 0.25.

The intercept *γ*_*i*_, the side effect *β*, and the digit effects *δ* are person-specific. This means that for these parameters, the model will produce one estimate for each participant. The multilevel model assumes that these individual-level effects in turn follow a normal distribution:

2 $${\displaystyle \begin{array}{rr}{\gamma}_i& \sim \mathrm{Normal}\left({\mu}_{\gamma },{\sigma}_{\gamma}^2\right),\\ {}{\beta}_i& \sim \mathrm{Normal}\left({\mu}_{\beta },{\sigma}_{\beta}^2\right),\\ {}{\delta}_{7i}& \sim \mathrm{Normal}\left({\mu}_{\delta_7},{\sigma}_{\delta_7}^2\right),\\ {}{\delta}_{6i}& \sim \mathrm{Normal}\left({\mu}_{\delta_6},{\sigma}_{\delta_6}^2\right),\\ {}{\delta}_{4i}& \sim \mathrm{Normal}\left({\mu}_{\delta_4},{\sigma}_{\delta_4}^2\right),\\ {}{\delta}_{3i}& \sim \mathrm{Normal}\left({\mu}_{\delta_3},{\sigma}_{\delta_3}^2\right).\end{array}}$$

The means (*μ*_*γ*_, *μ*_*β*_, *μ*_*δ*_) of these normal distributions represent the general expected effect; for example, *μ*_*β*_ represents the expected effect of digit side across individuals. The variances (${\sigma}_{\gamma}^2$, ${\sigma}_{\beta}^2$, ${\sigma}_{\delta}^2$) represent the individual variation. Therefore, the individual level depends on another level, the overall effect level, containing *μ*_*γ*_, *μ*_*β*_, *μ*_*δ*_, ${\sigma}_{\gamma}^2$, ${\sigma}_{\beta}^2$, and ${\sigma}_{\delta}^2$.

### Log-normal model

A normal model may not be appropriate in the case of more complex theories, hypotheses, or data structures. For example, the data in the example are response times. Response times cannot be negative. The normal model, however, can yield negative values. When using the normal model for response times, we are assigning probability to negative values that are not possible. This could be problematic. In addition, RT distributions are right-skewed. A better representation of the data at hand is therefore given by a log-normal model (Schramm & Rouder, 2019). This means that Eq. (1) has to be adjusted:

3 $${Y}_{ijk}\sim \mathrm{LogNormal}\left({\gamma}_i+{x}_j{\beta}_i+{u}_j{\delta}_{7i}+{v}_j{\delta}_{6i}+{w}_j{\delta}_{4i}+{z}_j{\delta}_{3i},{\sigma}^2\right).$$

Note that the only change in the model formulation is the change from a normal distribution to a log-normal distribution. The density function describes the probability model that is assumed to underlie the data. When the parameters of this probability model are known, the joint probability of the data can be inferred from the density function. The log-normal distribution only assigns probability to positive data values, and is, just as the RT distributions, right-skewed. Therefore, the log-normal distribution is a better match for the RT data. The location parameter of the log-normal distribution is described through the same combination of parameters as the mean of the normal model. However, it has a different interpretation. For example, for a location parameter of *μ* = 0 and a scale parameter of *σ*^2^ = 1, the mode of the log-normal distribution is 0.368, and the mean is 1.65.

### Summary

In summary, the models are placed directly on the raw RT data. The only difference between the normal and the log-normal models is the probability distribution. The normal model just assumes a symmetric normal distribution that can take on any value. The log-normal model assumes a log-normal distribution, which is restricted to positive values and has a right skew.

**Fig. 2:**
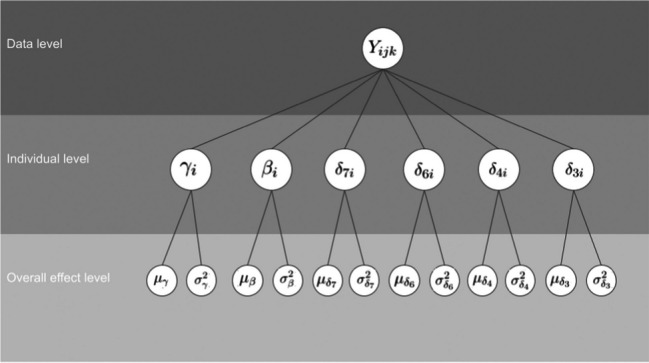
Hierarchical structure of the normal and log-normal models for the digit classification task

Both the normal and the log-normal models contain the following parameters: *γ*_*i*_ corresponds to the participant’s overall response time, *β*_*i*_ corresponds to the participant’s side effect, and the four *δ*_·*i*_ correspond to the effects between the digits furthest away from 5 (2 or 8) and the other digits. These person-specific effects all come from parent distributions. This results in the hierarchical structure shown in Fig. 2. The figure highlights the relationship between the parameters and the data.

Up to now, we have specified models without a correlation between the individual effects. This means, for instance, that we do not expect the estimations for the individual effects *δ*_7, *i*_ and *δ*_3, *i*_ to be related. However, in cases where we would expect the estimation of individual effects to be related, we could estimate the correlation coefficient between individual effects. This would add complexity to the model. For the sake of the tutorial, we will focus on the models without individual effect correlation, but we illustrate the estimation of the model with correlation between the individual effects in Online Supplement C and D.

In Bayesian modeling, prior distributions are needed to complete model specification. In the next section, we will take you through the process of setting these priors, followed by sections on Bayesian estimation and hypothesis testing for both the normal and log-normal models. In every section, we will first explain the normal model, followed by an illustration of the log-normal model.

## Prior settings

Bayesian analysis requires the specification of prior distributions. Prior distributions are probability distributions on model parameters that specify beliefs about the relative plausibility of parameter values before seeing the data (Wagenmakers et al., 2018). These prior beliefs can then be updated with the data to obtain the posterior beliefs about the model parameters, following Bayes’ rule (Jeffreys, 1961).

For our models, priors are needed on the general effects (*μ*_*γ*_, *μ*_*β*_, *μ*_*δ*_) and on the variances (${\sigma}_{\gamma}^2$, ${\sigma}_{\beta}^2$, ${\sigma}_{\delta}^2$, and *σ*^2^), as shown in Eq. (2). For each of these parameters, we specify our beliefs as a probability distribution. For example, if we expect small side effects in the digit classification example, the prior distribution on *μ*_*β*_ should have its peak close to zero. Large values for the variance parameters indicate large individual differences, whereas values close to zero indicate that effects are similar across individuals (Haaf & Rouder, 2017). If we expect little variability of side effects between people, the prior assigned to *σ*_*β*_ should also have its peak close to zero. The width of prior distributions expresses how certain we are about the parameters before seeing the data. For example, if we want to express that we are certain that the side effect is zero, we can formulate a prior that allocates all probability mass to a parameter value of zero.

### Fear of commitment

In many cases researchers fear committing to prior settings because they are unsure about their choices for the type and settings of the distribution. Therefore, they tend to choose wide priors to indicate little prior knowledge about the parameter values (Aczel et al., 2018). These include default priors offered by programming packages, such as *rstan* (Stan Development Team, 2019a; illustrated in Online Supplement B) and *brms* (Bürkner, 2017, 2018). Wide priors are priors that allocate (approximately) equal probability density to a great range of values, such as a uniform distribution without bounds (Gelman, 2006). However, wide priors can be problematic. First, since the distribution is spread out across a wide range of values, the probability density on any specific parameter value is very low. Therefore, any effect is unlikely under the prior, as a wide range of effects is deemed plausible. This influences model comparisons: the support for the null hypothesis becomes increasingly large. This is also referred to as the Jeffreys–Lindley paradox (Lindley, 1957).

**Fig. 3:**
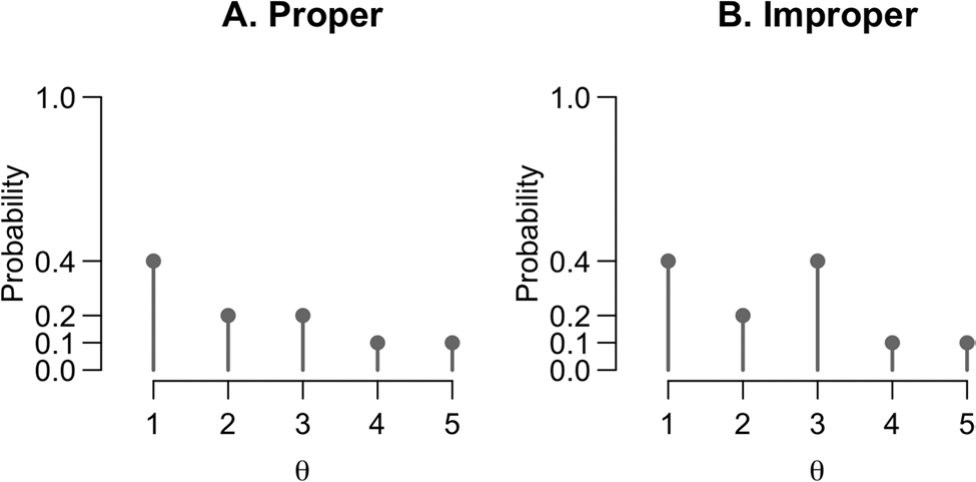
Two hypothetical priors for a discrete parameter *θ*. **A** A proper probability distribution that sums to 1. **B** An improper probability distribution that sums to 1.2

Another important issue is that wide priors are often improper probability distributions (Hobert & Casella, 1996). A prior is proper if two conditions hold: (1) All values of the probability distribution are equal to or greater than zero, and (2) the probability distribution sums to 1 for discrete data or integrates to 1 for continuous data. An example of a proper and an improper probability distribution are provided in Fig. 3. The figure shows two hypothetical priors for parameter *θ*. The first condition can be assessed by checking the *y*-values—both priors have only positive values as function output (0.1, 0.2, and 0.4). To assess the second condition, we need to sum up the function values. Panel A shows a proper distribution where the values sum to 1; panel B shows an improper distribution where the values sum to 1.2.

A more common example of an improper prior is a uniform prior distribution ranging from minus infinity to infinity. In the case of improper priors, it is often impossible to obtain correct estimation of general and individual effects in hierarchical models because the resulting posteriors will again be improper probability distributions. However, even proper priors can result in improper posteriors (Hobert & Casella, 1996). Although estimation should not be possible in this situation, statistical software might still provide results without notifying the user that the posterior distribution does not exist. If users are not aware of the impropriety problem, this can result in misleading conclusions. Therefore, it is important to check whether (a) the priors are proper and (b) the priors result in proper posteriors. Unfortunately, there is no fail-safe method that guarantees proper posteriors for hierarchical models. The best approach is to choose well-reasoned informative priors for the model, for instance, by using the proposed four-step procedure below. Priors that are proper and not too diffuse will in most cases lead to proper posteriors.

### Procedure

**Fig. 4:**
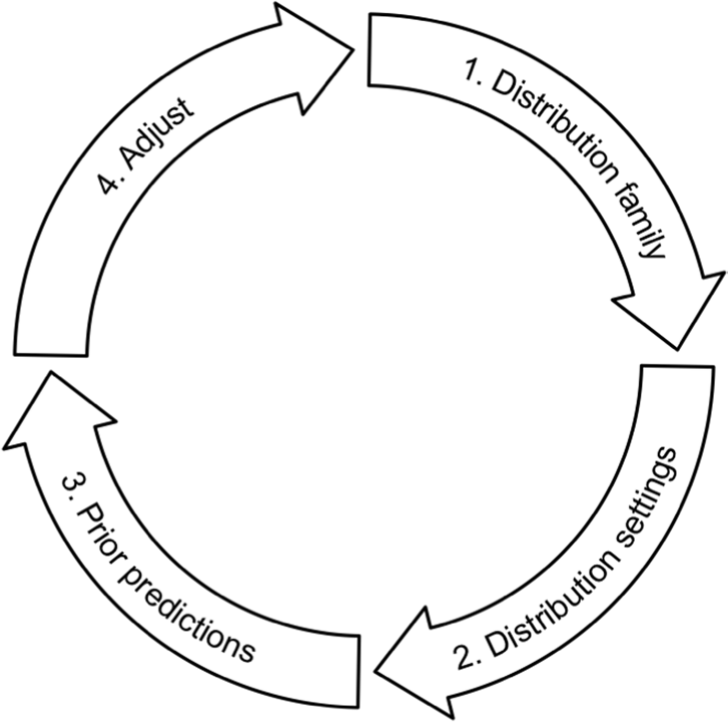
Prior specification steps

We propose four steps for choosing suitable priors for Bayesian hierarchical models (see Fig. 4). We first present the steps and then apply them to choose suitable priors for the parameters *μ*_*γ*_, *μ*_*β*_, *μ*_*δ*_, ${\sigma}_{\gamma}^2$, ${\sigma}_{\beta}^2$, ${\sigma}_{\delta}^2,$ and *σ*^2^ in our example.

### Step 1: Distribution family

**Fig. 5:**
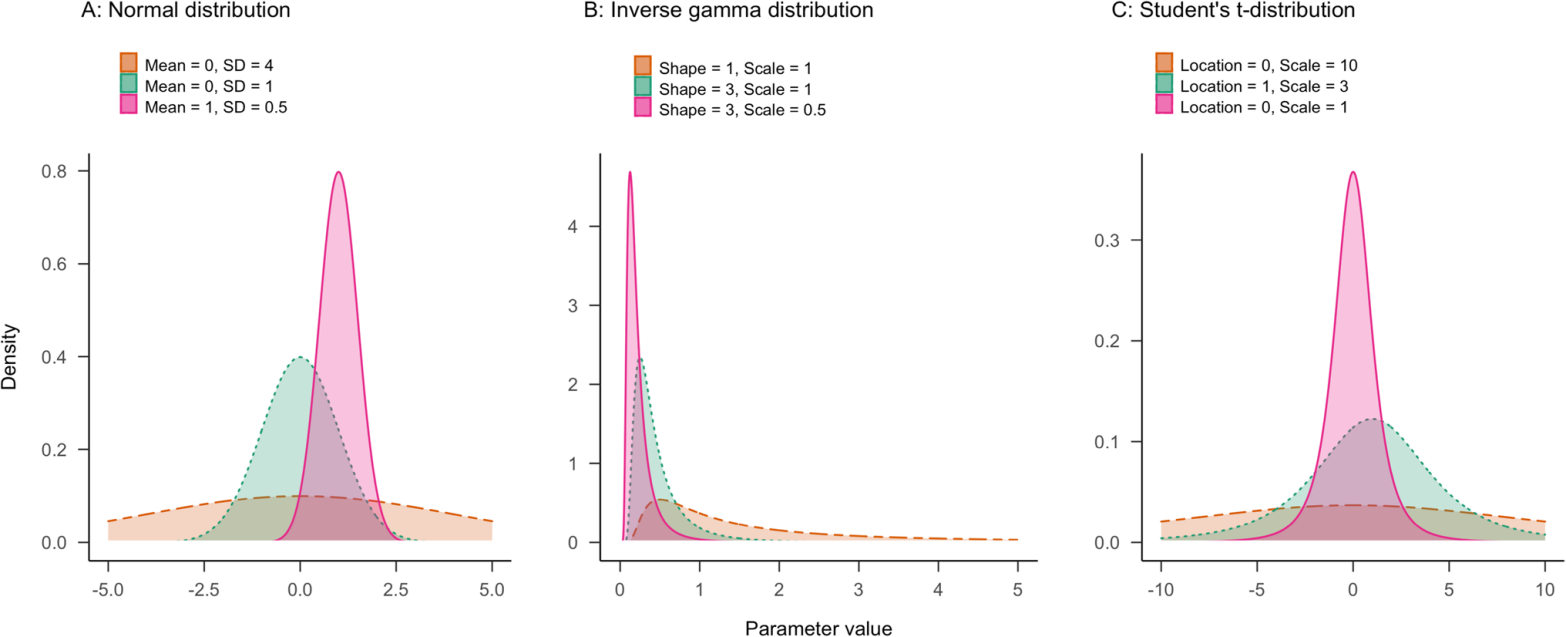
Common probability density distributions that may be used as priors. Panel **A** shows normal distributions varying in mean and standard deviation. Panel **B** shows inverse gamma distributions that differ in the shape and scale parameter. Panel **C** shows Student’s *t*-distributions that differ in the location and scale parameter. For all three *t*-distributions, the degrees of freedom are set to 3

The first step in specifying a prior distribution is to find a suitable distribution family. For instance, the prior plausibility of parameter values can be expressed through a normal, an inverse gamma, or a Student *t*-distribution. These distributional families have different shapes and a different support, as illustrated in Fig. 5. For example, the inverse gamma distribution is limited to non-negative parameter values and has a positive skew. The normal and Student *t*-distribution both have support over the real line; however, the Student *t*-distribution has wider tails, that is, it assigns a higher plausibility to values that are on the extremes of the distribution.

The support of the prior distribution should cover all theoretically possible parameter values. For example, if a parameter cannot have negative values, such as a standard deviation, the inverse gamma distribution would be a suitable choice. Conversely, if a parameter can take positive and negative values, the normal and Student *t*-distribution would be suitable.

Note that although distributions within a distributional family share the same functional form, they can differ considerably in their shape and location. The exact appearance of a prior distribution is controlled by a set of hyperparameters, for example, the mean and variance for the location and scale of a normal prior. In the next section, we will give some guidance for specifying these hyperparameters.

### Step 2: Set hyperparameters

The location, scale, and sometimes shape of the selected distribution can be adjusted by choosing adequate hyperparameters. The goal is to adjust the prior to reflect which parameter values we deem most plausible and how much uncertainty there is. For instance, panel A of Fig. 5 illustrates that increasing the mean in the normal distribution leads to a shift of location: higher parameter values are deemed more plausible according to the pink distribution than the green distribution. Panel B shows how uncertainty can be specified, for example by decreasing the shape of the inverse gamma distribution while keeping the scale constant: the orange distribution assigns probability to a wider range of values than the green distribution.

For this step, it is important to consider the scale on which variables are measured. For instance, RTs could be measured in seconds or in milliseconds. This means that if a researcher expects a response time of one second, they will formulate a prior distribution with a mean of 1 if RT is measured in seconds, or formulate a prior distribution with a mean of 1000 if RT is measured in milliseconds. Similarly, the measurement scale will also affect the width of the specified prior.

### Step 3: Prior predictions

A key advantage of model specification with priors is that we can obtain predictions on what the data generated from the model would look like (Etz et al., 2018; Lee & Vanpaemel, 2018). These prior predictions allow us to make the implications of our specified priors more concrete. Researchers can look at relevant statistics that summarize the simulated data and decide whether they correspond to reasonable observations for their specific research context. If the simulated data are in line with the researchers’ expectations, the priors can be used for the analysis. If the simulated data do not match the expectations, the priors have to be adjusted (step 4).

**Fig. 6:**
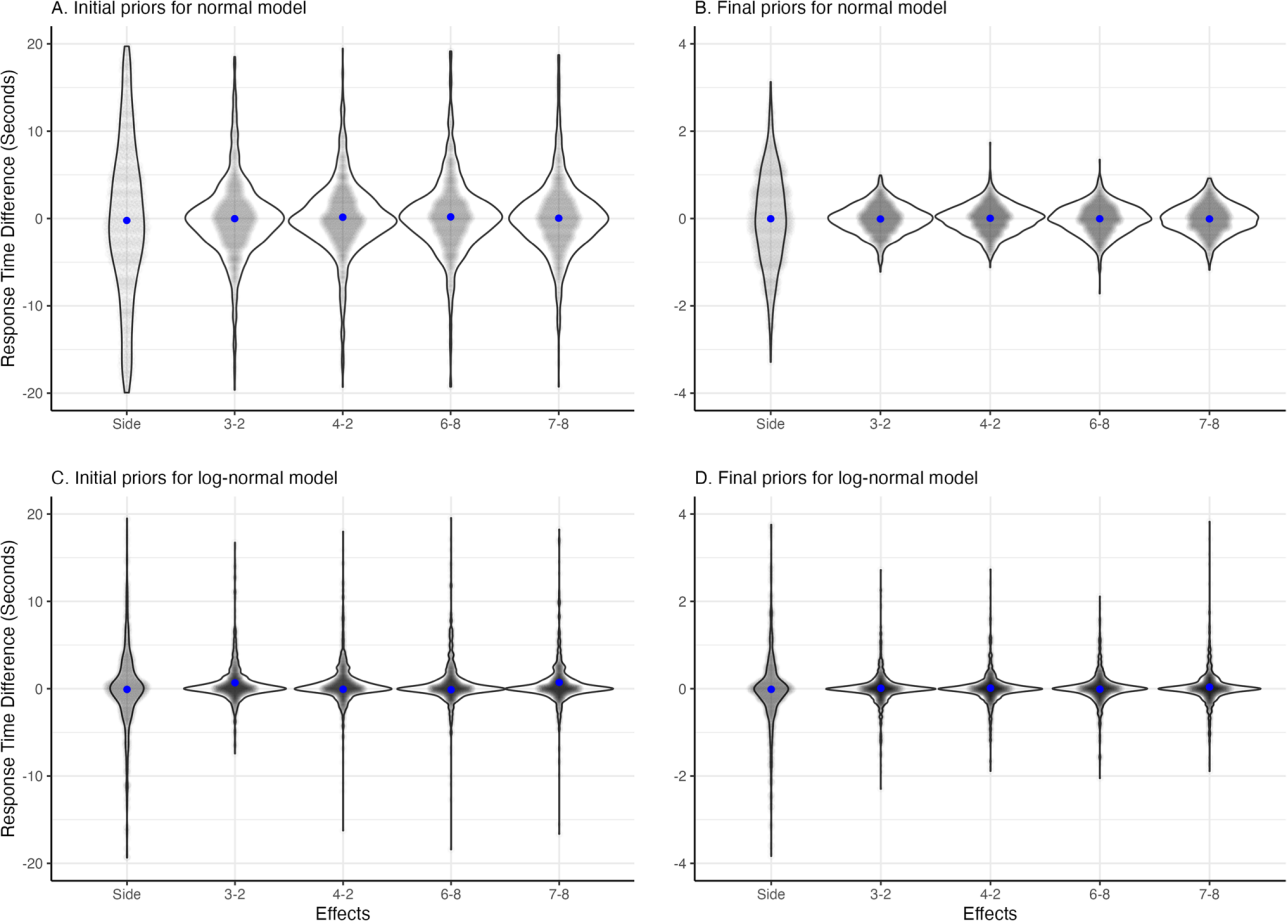
Side and digit effects from predicted data. Violins represent the distribution of predicted effects across 1000 simulation runs. The blue points are the average effects across simulations. Panel **A** shows the prior predictions for the normal model based on the start priors. Panel **B** shows the prior predictions for the normal model based on the selected priors. Panel **C** shows the prior predictions for the log-normal model based on the priors chosen for the normal model. Panel **D** shows the prior predictions for the log-normal model based on the selected priors

The simulation proceeds as follows: First, one random draw from each prior distribution is taken. This is plugged into the individual effect distributions [Eq. (2)]. Then, the probability of the model, such as specified in Eqs. (1) and (3), is used to simulate observations per condition. This means that for *n* participants, *n* random values for the model parameters are generated based on the priors, which will result in the *k* observations per *j* conditions. This process is repeated *m* times, where *m* is the number of simulations drawn. Note that the greater the number of simulations conducted, the closer one gets to the full picture because a greater number of samples are available from the prior distribution. Finally, we can visualize the simulated data. Figure 6 shows prior predictions for the key effects from the digit classification task. For this task, we mainly care about the side effect and digit effects corresponding to the *β*s and the *δ*s from the model. Here, the prior predictions for the average side effect and the average digit effect across participants are depicted.

For step 3, the intention is not to compare the actual data of interest to the data predicted by the priors. Instead, the researchers’ *expectations* of plausible data are compared to the prior predictions. Therefore, prior predictive checks are conducted *before* looking at the actual data. If the prior predictions match the expectations, the priors can be used for the analysis. If they do not match the expectations, the priors need to be adjusted.

### Step 4: Adjust

Based on the results of the prior predictions, it might be necessary to adjust the priors. Typically, the focus of adjustments will be on finding adequate parameter values for the prior distribution, that is, on repeating step 2 and the following steps of the prior specification process.

In the following two sections, we will provide a walk-through of all four steps of prior specification for our two models of the symbolic distance effect.

### Normal model

In the symbolic distance model, we have to specify priors for the parameters *μ*_*γ*_, *μ*_*β*_, *μ*_*δ*_, ${\sigma}_{\gamma}^2$, ${\sigma}_{\beta}^2$, and ${\sigma}_{\delta}^2$. The parameters *μ*_*γ*_, *μ*_*β*_, and *μ*_*δ*_ can be positive or negative. All variance terms (${\sigma}_{\gamma}^2$, ${\sigma}_{\beta}^2$, ${\sigma}_{\delta}^2$, and *σ*^2^) are constrained to be positive. If there is no information from previous studies, researchers might be inclined to use the default priors of software packages, often reasonably uninformative priors, as a starting point.

We take the reasonably uninformative priors as a starting point as well as specifying priors on different types of parameters. These priors were previously used as default priors in *brms* (Bürkner, 2017). The prior on the group-level regression weights (*μ*_*β*_, *μ*_*δ*_) is a normal distribution with a mean of 0 and a standard deviation of 1. The prior on the intercept *μ*_*γ*_ is a scaled and shifted Student *t*-distribution with three degrees of freedom, a location parameter of 1, and a scale parameter of 10. *brms* specifies priors on standard deviations ($\sqrt{\sigma^2}$) instead of variances. For the normal model, this means that priors are set on *σ*_*γ*_, *σ*_*β*_, *σ*_*δ*_, and *σ*, instead of on ${\sigma}_{\gamma}^2$, ${\sigma}_{\beta}^2$, ${\sigma}_{\delta}^2,$ and *σ*^2^. The prior on the standard deviations is a central, scaled Student’s *t*-distribution with three degrees of freedom and a scale parameter of 10 that is truncated at zero. To summarize, the starting priors would be set the following way:

4 $${\displaystyle \begin{array}{rl}{\mu}_{\gamma }& \sim {\mathrm{Student}}^{'}\mathrm{s}\ t\left(3,1,10\right),\\ {}{\mu}_{\beta },{\mu}_{\delta }& \sim \mathrm{Normal}\left(0,1\right),\\ {}\sigma, {\sigma}_{\gamma },{\sigma}_{\beta },{\sigma}_{\delta }& \sim \mathrm{Truncated}-\mathrm{Student}'\mathrm{s}\ {t}_{+}\left(3,0,10\right).\end{array}}$$

The prior distributions specified on the standard deviations can be transformed to prior distributions on variances (*var*). Prior distributions on variances have a smaller scale than priors on standard deviation (*sd*). Note, however, that the transformation of the prior distributions is not as simple as the transformation of the parameter itself. If we formulate a prior on standard deviations, there is always an implied prior on the variances as well. If we think of the distribution as *f*(*x*), *x* can either be defined as $\sqrt{\left(\mathit{\operatorname{var}}\right)}$, if we formulated the prior on the standard deviation, or *x* can be defined as *sd*^2^ if we formulated the prior on the variance. From this, we can derive the implied distribution *f*(*var*) or *f*(*sd*). Notably, this is not the same as calculating $\sqrt{\left(f(x)\right)}$ or *f*(*x*)^2^. To transform the priors on standard deviations to variances, we try to find the parameters that match the prior distribution to the implied distribution. In Online Supplement J, we provide R code with a numeric solution to the transformation. For the *brms* priors, we obtain the following implied prior distributions on variance﻿s:

5 $${\displaystyle \begin{array}{rr}{\sigma}_{\gamma}^2,{\sigma}_{\beta}^2,{\sigma}_{\delta}^2,{\sigma}^2& \sim {\mathrm{Student}}^{'}\mathrm{s}\ t\left(3,0,1.48\right).\end{array}}$$

**Fig. 7:**
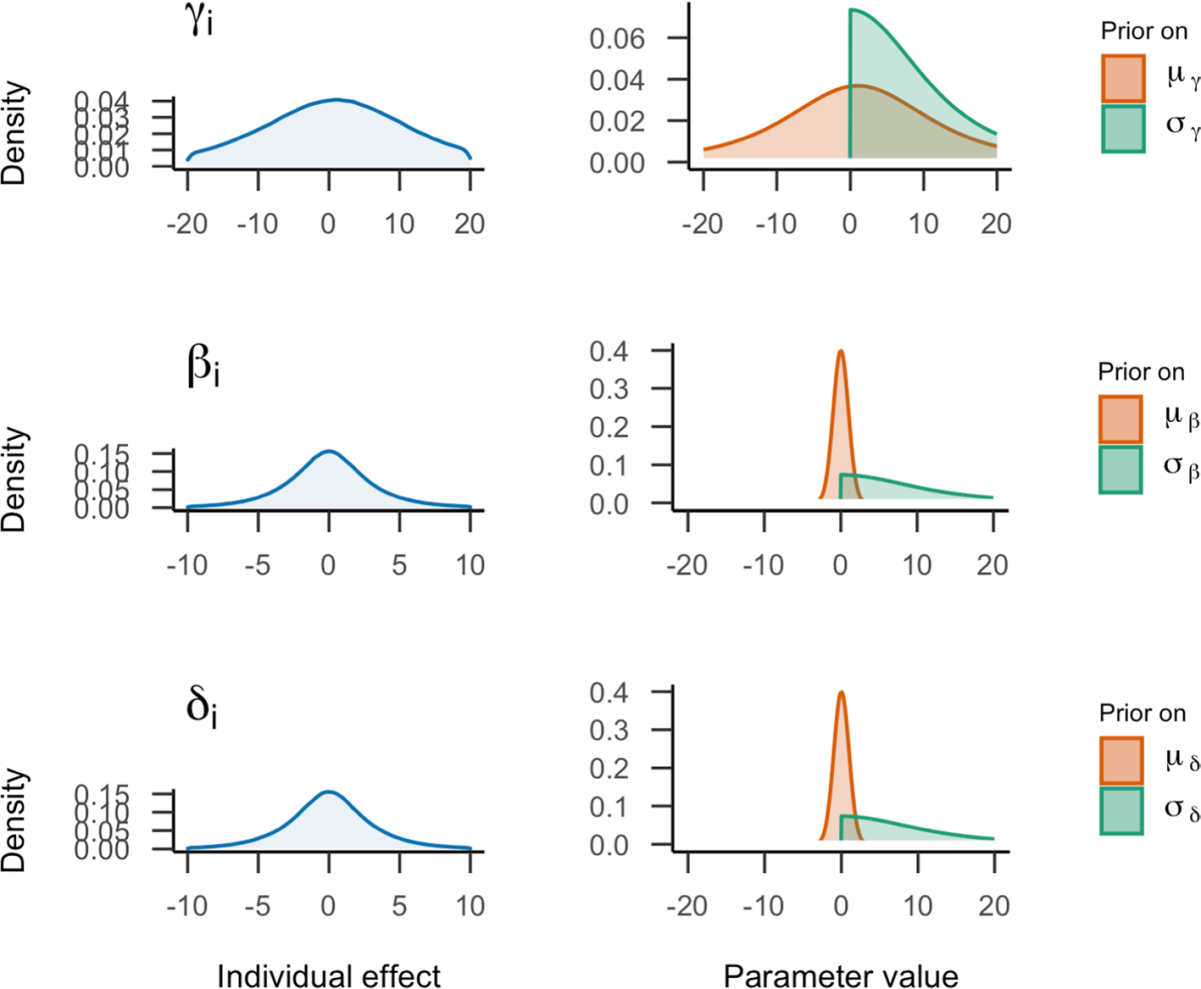
Visualization of starting prior settings for the normal model. On the right, the prior distributions on general effects parameters are shown. On the left, the implied distribution of the individual effects for every parameter based on these priors are presented

The priors are visualized on the right side of Fig. 7. Note that these prior distributions are on the group-level parameters and represent the uncertainty about the general effect. The uncertainty about individual effects is represented by the marginal prior distribution on the left. This distribution takes the prior variability of the mean and variance as well as the implied individual variability into account.

We will continue with step 3 of the prior specification process and check what the expected data would look like according to the selected priors. The results of the prior prediction for the symbolic distance effect with the priors from Eq. (4) with the number of participants *n* = 10, the number of trials per condition *k* = 60, and the number of simulations *m* = 1000 are shown in panel A of Fig. 6. Code for the simulations can be found in Online Supplement J.

The figure shows the difference in RTs between digits for every repetition averaged across subjects (i.e., 1000 gray dots per effect). The blue dots represent the mean effect. Using the reasonably uninformative priors, the mean effects are expected to be around zero, with a mean effect of zero. However, the range around this mean is very wide, ranging from −10 to 10 second effects. Most of the trials are in a smaller range of −5 to 5 second effects. This means that according to our priors, we would expect the effects to fall in this range. For this task, where participants have to respond as fast as possible, such large RT effects are unlikely. Therefore, it seems reasonable to adjust the priors.

The normal distribution on *μ*_*β*_ and *μ*_*δ*_ seems reasonable if we assume that a two-sided hypothesis about the symbolic distance effect is tested. However, we expect less variance around this mean. Therefore, we decrease the setting for the variation. The wide tails of the Student *t*-distribution could be one of the reasons for the high range in variation. We change the prior for *μ*_*γ*_ to a normal distribution that has more narrow tails. As RTs cannot be negative, we adjust the prior to be positive only by truncating the normal distribution using a lower bound of zero. Finally, we would like to use a more common conjugate prior for the variances to ensure that we obtain a proper posterior distribution.^2^,^3^ Therefore, we choose an inverse gamma distribution. We can also be more specific about the expected RTs. Based on our knowledge of these types of tasks (e.g., Haaf & Rouder, 2017), it seems highly plausible that participants will respond within a second or two. We would expect effects in the range of 10 milliseconds to 100 milliseconds. We adjust our priors using these new distribution types and settings, evaluate the prior predictions (shown in panel B of Fig. 6), and arrive at the following priors:^4^

6 $${\displaystyle \begin{array}{rl}{\mu}_{\gamma }& \sim \mathrm{Truncated}-{\mathrm{Normal}}_{+}\left(0.5,1\right),\\ {}{\mu}_{\beta }& \sim \mathrm{Normal}\left(0,0.09\right),\\ {}{\mu}_{\delta }& \sim \mathrm{Normal}\left(0,0.09\right),\\ {}{\sigma}^2& \sim \mathrm{Inverse}-\mathrm{Gamma}\left(3,0.7\right),\\ {}{\sigma}_{\gamma}^2& \sim \mathrm{Inverse}-\mathrm{Gamma}\left(3,0.7\right),\\ {}{\sigma}_{\beta}^2& \sim \mathrm{Inverse}-\mathrm{Gamma}\left(3,0.5\right),\\ {}{\sigma}_{\delta}^2& \sim \mathrm{Inverse}-\mathrm{Gamma}\left(3,0.5\right).\end{array}}$$

Setting equivalent prior distributions on the standard deviations instead of on the variances results in the following priors:

7 $${\displaystyle \begin{array}{rl}{\mu}_{\gamma }& \sim \mathrm{Truncated}-{\mathrm{Normal}}_{+}\left(0.5,1\right),\\ {}{\mu}_{\beta }& \sim \mathrm{Normal}\left(0,0.3\right),\\ {}{\mu}_{\delta }& \sim \mathrm{Normal}\left(0,0.3\right),\\ {}\sigma & \sim \mathrm{Inverse}-\mathrm{Gamma}\left(13.8,6.3\right),\\ {}{\sigma}_{\gamma }& \sim \mathrm{Inverse}-\mathrm{Gamma}\left(13.8,6.3\right),\\ {}{\sigma}_{\beta }& \sim \mathrm{Inverse}-\mathrm{Gamma}\left(13.8,5.3\right),\\ {}{\sigma}_{\delta }& \sim \mathrm{Inverse}-\mathrm{Gamma}\left(13.8,5.3\right).\end{array}}$$

**Fig. 8:**
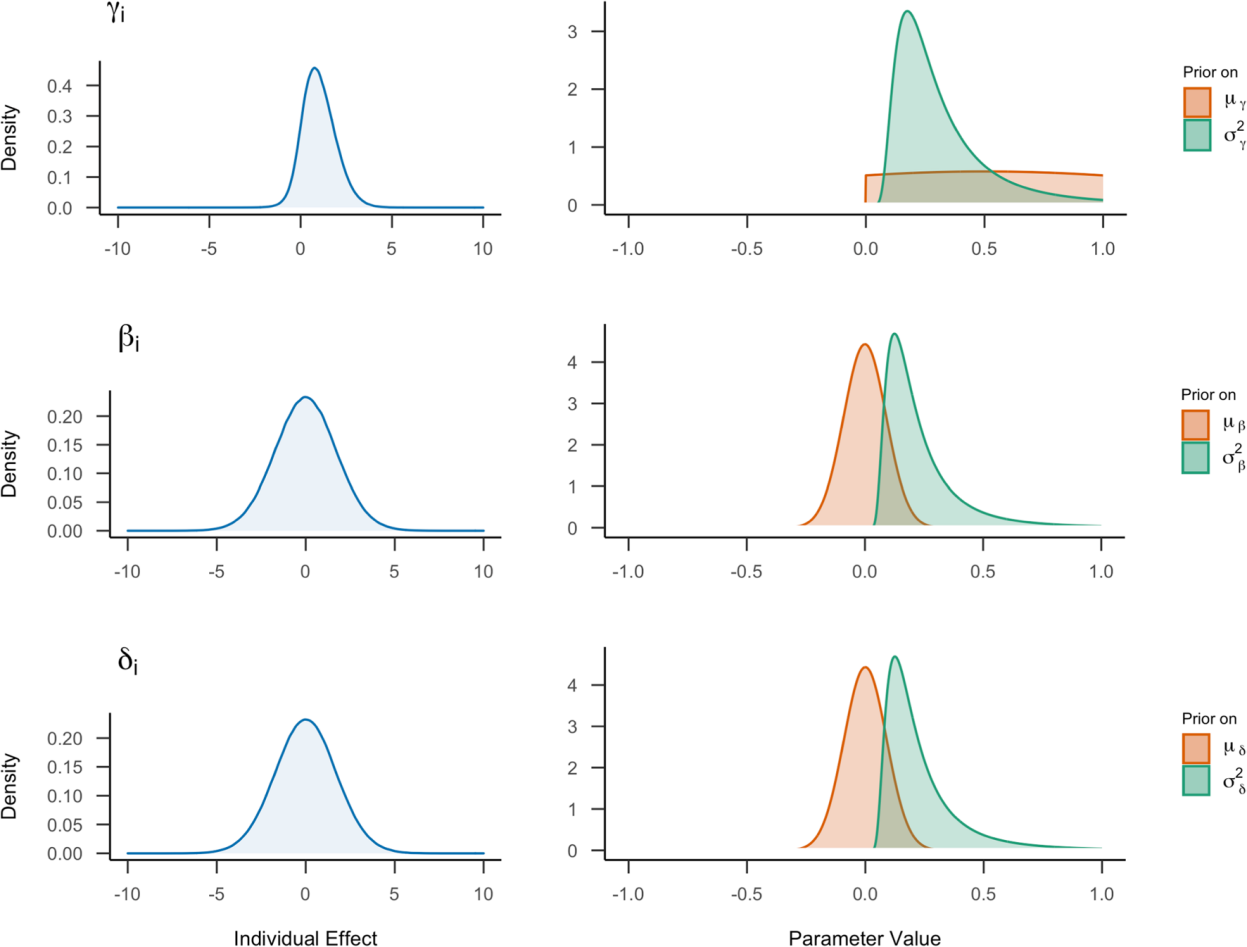
Visualization of the adjusted prior settings for the normal model. On the right, the prior distributions on general effects parameters are shown. On the left, the implied distribution of the individual effects for every parameter based on these priors is presented

The adjusted priors are visualized in Fig. 8. The figure shows that the adjusted priors assign their highest probability density to a smaller range of values and are, therefore, more specific than starting priors presented in Fig. 7. In addition, the shape of the distribution is different for the individual variation, resulting in a higher probability for values closer to zero. Panel B of Fig. 6 shows the predictions of the normal model with adjusted prior distributions. Most effects now range between −0.5 and 0.5 seconds. This aligns better with our expectations based on existing literature, but still allows for a considerable uncertainty about the true size of the effects.

### Log-normal model

We follow the same procedure for prior specification in the log-normal model. As before, we prefer to apply conjugate priors. The conjugate priors that we applied to the normal model are also conjugate priors for the log-normal model (Fink, 1997b). For instance, with a normal distribution as prior for the mean of the log-normal model, we assume that the mean of the parameter can be negative, zero, or positive, and assign a higher probability to values closer to zero. If the mean of the log-normal distribution is smaller than zero, this means that the peak of the log-normal distribution moves closer to zero, i.e., that response times are quicker but still larger than zero. When applying the normal distribution as prior, the posterior of the mean in the log-normal model will result in a normal distribution as well. As a starting point, we can use the adjusted priors of the normal model and perform a prior prediction, shown in panel C of Fig. 6. These priors result in a slightly higher variance in RTs than we would expect, and, importantly, the mean response time differences are not centered around zero in all conditions. This pattern is inconsistent with our expectations, which means that prior distributions for the log-normal model need to be adjusted.

The transformation of the prior distributions is not as simple as the transformation of the parameter itself. Panel C shows that with the same priors as for the normal model, we obtain different data for the log-normal model compared to the normal model (panel B). Therefore, we adjusted the priors until the simulated data matched our expectations and looked like panel B. This yields the following priors that result in the prior prediction shown in panel D of Fig. 6:^5^

8 $${\displaystyle \begin{array}{rl}{\mu}_{\gamma }& \sim \mathrm{Normal}\left(-0.5,1\right),\\ {}{\mu}_{\beta }& \sim \mathrm{Normal}\left(0,0.005\right),\\ {}{\mu}_{\delta }& \sim \mathrm{Normal}\left(0,0.005\right),\\ {}{\sigma}^2& \sim \mathrm{Inverse}-\mathrm{Gamma}\left(3,0.3\right),\\ {}{\sigma}_{\gamma}^2& \sim \mathrm{Inverse}-\mathrm{Gamma}\left(3,0.3\right),\\ {}{\sigma}_{\beta}^2& \sim \mathrm{Inverse}-\mathrm{Gamma}\left(3,0.01\right),\\ {}{\sigma}_{\delta}^2& \sim \mathrm{Inverse}-\mathrm{Gamma}\left(3,0.01\right).\end{array}}$$

Setting the same prior distribution on the standard deviations instead of on the variances yields the following priors:

9 $${\displaystyle \begin{array}{rl}{\mu}_{\gamma }& \sim \mathrm{Normal}\left(-0.5,1\right),\\ {}{\mu}_{\beta }& \sim \mathrm{Normal}\left(0,0.07\right),\\ {}{\mu}_{\delta }& \sim \mathrm{Normal}\left(0,0.07\right),\\ {}\sigma & \sim \mathrm{Inverse}-\mathrm{Gamma}\left(13.8,4.1\right),\\ {}{\sigma}_{\gamma }& \sim \mathrm{Inverse}-\mathrm{Gamma}\left(13.8,4.1\right),\\ {}{\sigma}_{\beta }& \sim \mathrm{Inverse}-\mathrm{Gamma}\left(13.8,0.7\right),\\ {}{\sigma}_{\delta }& \sim \mathrm{Inverse}-\mathrm{Gamma}\left(13.8,0.7\right).\end{array}}$$

### Prior sensitivity

In the previous sections, we decided on one set of prior distributions for each of the proposed models. However, researchers may find it difficult to settle on a single set of prior distributions in practice. This raises the question: What happens if you cannot decide on one prior? If several possibilities seem plausible, it is possible to use them all separately for the analysis and investigate their influence on the analysis results. This is called a prior sensitivity analysis (Roos et al., 2015). Generally, when it comes to prior specification, it is important to be *transparent* and to provide a justification for the selected prior distributions (Stefan et al., 2022). Prior distributions are rarely inadmissible. However, as we demonstrated earlier, their usefulness in constraining model predictions can vary. Therefore, providing a justification of prior distributions in a paper can help readers gauge the generative quality of a presented model. When multiple justifiable prior distributions are applied to the same analysis, it is important to be transparent about this to avoid the impression of cherry-picking priors. If priors are chosen for their best fit to the data, this can lead to overfitting and spurious results. Prior sensitivity analyses, and transparency about the prior specification process more generally, can therefore help to increase trust in the research results.

## Bayesian parameter estimation

In Bayesian statistics, parameter estimates are obtained from the posterior distribution. The posterior distribution captures the uncertainty regarding the parameter after seeing the data, **Y**. The mean of this distribution can be used as a point estimate for the parameter. The posterior distribution is calculated by updating the prior distribution using Bayes’ rule. For the symbolic distance effect, we have a model with many parameters, so the posterior distribution is also multidimensional. Instead of writing out all the parameters, we replace all the model parameters with the vector **θ**, resulting in the joint posterior:

10 $$\underset{\mathrm{P}{uposterior}}{\underbrace{\mathrm{P}\left(\boldsymbol{\uptheta} |\textbf{Y}\right)}}=\underset{\text{Prior}}{\underbrace{\mathrm{P}\left(\boldsymbol{\uptheta} \right)}}\times \underset{\text{Updating factor}}{\underbrace{\frac{\mathrm{P}\left(\textbf{Y}|\boldsymbol{\uptheta} \right)}{\mathrm{P}\left(\textbf{Y}\right)}}}.$$

Typically, we are not interested in the distribution of all parameters together, but in the posterior of one parameter independent of all the others. This posterior distribution is called the marginal posterior, and it is computed by integrating out all other parameters of the joint posterior. The marginal posterior distribution for the side effect *μ*_*β*_, for instance, is:

11 $$\mathrm{P}\left({\upmu}_{\upbeta}|\textbf{Y}\right)=\int \textbf{P}\left(\textbf{Y}|{\upmu}_{\upbeta},\boldsymbol{\uptheta} \right)\textbf{P}\left({\upmu}_{\upbeta}\right)\textbf{P}\left(\boldsymbol{\uptheta} \right)\textbf{d}\boldsymbol{\uptheta}$$

where **θ** now represents all parameters except for *μ*_*β*_. With an increasing number of parameters and an increasingly complex model structure, it is no longer possible to find analytical solutions for the multidimensional integrals involved in the posterior distributions. A solution to this issue is to sample from the joint posterior distribution using algorithms such as Markov chain Monte Carlo sampling (MCMC; for an introduction see van Ravenzwaaij et al., 2018), Gibbs sampling (Chib, 1995), or Hamiltonian Monte Carlo sampling (Betancourt & Girolami, 2015). It is beyond the scope of this paper to go into detail on the algorithms, but we refer the interested reader to a tutorial paper on MCMC by van Ravenzwaaij et al. (2018), a conceptual introduction to Hamiltonian Monte Carlo by Betancourt (2018), and a summary of samplers by Green et al. (2015).

An approximation for the marginal posterior distribution can be obtained by assessing the empirical distribution of MCMC samples for the parameter of interest, disregarding the other parameters. Since the frequency of combinations of samples in the joint posterior is determined by their posterior plausibility, this procedure is equivalent to a multidimensional integration across all other parameters.

Every sampling algorithm requires the specification of the number of samples (also called iterations) that are drawn from the posterior distribution. Furthermore, all MCMC sampling methods have in common that results depend (somewhat) on chosen starting values, that is, initial parameter values that are fed to the sampler in the first iteration. This dependence can be assessed if the sampling algorithm is repeated several times (i.e., chains) with different starting values. Since the first few iterations of a chain—often called warm-up or burn-in—are somewhat dependent on the starting value, these samples are typically discarded.

Before interpreting the estimation results, one has to check whether the posterior distribution of the parameters has converged, meaning that a stationary posterior distribution has been reached (Vehtari et al., 2021). We will explain these convergence checks in more detail in the “Model diagnostics” section.

### Software

There are many different programs available to fit Bayesian hierarchical models, that is, to obtain samples from the joint posterior distribution of parameters. In this tutorial, we focus on two of the most commonly used R packages, *rstan* (Stan Development Team, 2019a) and *brms* (Bürkner, 2017, 2018). We assume that readers are somewhat familiar with R. The package *rstan* provides the most modern algorithms and flexibility in model setup. The package *brms* was created as an overlay to *rstan* and is based on the well-known *lme4* syntax (Bates et al., 2015). The goal of *brms* is to ease the transition to Bayesian hierarchical modeling for novice users of Bayesian statistics. The packages work similarly for the normal and log-normal models. In this section, we explain how a model can be fitted in each package using code snippets. In addition, we offer separate R Markdown files for each package in Online Supplements C–F that explain in more depth how to analyze the full symbolic distance effect model with the specific package.

#### rstan

*rstan *allows the application of Stan (Carpenter et al., 2017), a probabilistic programming language, in R. The package uses a version of the Hamiltonian Monte Carlo (HMC) algorithm, No-U-Turn Sampler (NUTS; Hoffman & Gelman, 2014), for sampling. *rstan* requires two files: a .*stan* file containing the model specification and an R script containing the code for the model fitting. In the *.stan* file, the parameters, priors, and the probability distribution of the data are specified. An example *.stan* file of a simple, non-hierarchical normal model, where the variance is already known and the mean has to be estimated, is shown below (adapted example from Nicenboim et al., 2021, Chapter 10). The file is divided into three sections: data, parameters, and model.

 The data section specifies the variables in the data set, as well as constant values in the model. For the symbolic distance effect model, the data section contains the total number of observations, the participant index variable, the response time variable in the data set, the condition for every response time [side of the digit and the digit indicator, i.e., variables *x*_*j*_, *u*_*j*_, *v*_*j*_, *w*_*j*_, and *z*_*j*_ from (1)], and the prior specification according to Eqs. (6) and (8). The parameters section specifies all parameters that are estimated. These are the general and individual effects. The last section, model, contains the probability function and priors, as specified earlier in this article. Instead of using *y[i] ~ normal(mu, sigma)* to estimate parameters, such as the mean from the example, we used the target specification (for an in-depth explanation see Nicenboim et al., 2021, Chapter 10). Both methods indicate in this case that *y[i]* is normally distributed with the mean *mu* and the variance *sigma*. However, the target method saves constant terms that are required for model comparison (Gronau et al., 2020) The *.stan* file for the symbolic distance effect, including explanations, can be found in Online Supplement K.

In the corresponding R file, the model is fitted to the data using the stan function, as shown below. In this function, it is possible to specify the number of iterations, chains, and warm-up samples in the Monte Carlo sampling procedure. Next to these specifications, the function requires a directory path to the *.stan* file and an R object of type list containing the data (here called *myData_list*). Every element of the data section in the *.stan* file needs to be specified with the same name in the data object.

 The package *rstan* offers considerable flexibility. It is possible to specify the probability model and priors exactly as wished. However, this comes with the price of some technical knowledge. The commented code for fitting the full symbolic distance effect model using *rstan *can be found in Online Supplement C: *Bayesian Hierarchical Modeling in rstan*.

#### brms

The R package* brms* also allows for fitting Bayesian models using Stan. However, model specification and model fitting can be achieved using a much simplified R script alone. Model specification is based on a formula syntax that is similar to the notation in the popular *lme4* package for frequentist hierarchical modeling (Bates et al., 2015). The formula is an object that specifies the dependent variable as a function of the independent variables. The ~ (tilde) separates the dependent variable (*rt*) on the left side from the independent variables on the right side. The formula distinguishes between general effects and individual deviations. The first part of the equation on the predictor side of the formula shown below (i.e., *1 + side + dif1 + dif2 + dif3 + dif4*) represents the general effects. The *1* represents the intercept, in the case of the symbolic distance effect *μ*_*γ*_, and side and *dif1* to *dif4* represent the general effects of side and distance (*μ*_*β*_ and *μ*_*δ*_). Within the round brackets individual deviations and grouping variables are specified. In this case the grouping variable (*ind*) indicates that each participant has an individual effect. In other cases effects might vary per item, block, or even the combination of participant and item. The double bar (||) indicates that the correlation between the parameters should not be modeled. When using a single bar (|), then correlations across all individual effects (intercept, side, and distance) are modeled. This approach, however, is a bit more difficult to interpret and would also not correspond to the implemented Stan model.

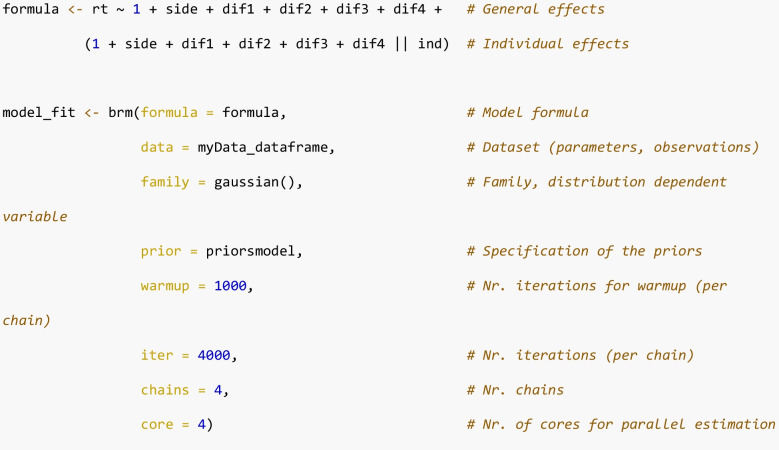



The brm function requires a data frame (here called *myData_dataframe*) that contains all variables mentioned in the formula. The argument family in the *brm* function specifies the exponential family of distributions according to which the dependent variable is assumed to be distributed. For the normal model, this argument takes the value *gaussian*; for the log-normal model, it needs to be specified as *lognormal*.

Finally, we specify the priors. In *brms*, priors can be set on the general effects represented by *class = b* and on the individual variation represented by *class = sd*. It is important to note that priors can only be set on the specific parameters that are part of the *brms* model specification. For example, *brms* only allows setting priors on standard deviations and not on variances. Therefore, for the normal model of the symbolic distance effect, we will use the priors for standard deviations specified in Eq. (7). To set the priors, we use the *set_prior* function, as shown below. In this function, first, the distribution is specified, using the Stan programming language. Then, the type of parameter to which the prior applies is defined by *class*. The parameter to which this prior applies is specified by *coef*. For the priors on the individual effects, an additional element has to be specified, namely, the grouping variable (i.e., *group*). For example, the prior distributions on the general side effect, *μ*_*β*_, and on its variability between individuals, *σ*_*β*_, can be specified as follows:

 If you do not specify a prior for a certain parameter, the default prior as explained in the prior section will be applied. It is possible to save all priors in an R *data.frame* and provide these in the *brm* function. This and the full code with explanation for fitting the full symbolic distance effect model using *brms* are shown in our Online Supplement D: Bayesian Hierarchical Modeling in brms.^6^

## Estimation of the digit classification task

### Data

For our example, we will use the data set by Rouder et al. (2005). The data are available under a free license on GitHub:  https://github.com/PerceptionCognitionLab/data0/tree/master/lexDec-dist5. The data set contains observations from 54 participants who took part in a digit classification task. Each participant performed 360 trials in six blocks. For the analysis, only the correct responses are included. To account for practice effects, the first 25 trials of the first block are removed from the analysis. The trials after the breaks between the blocks are also removed from the analysis to correct for lack of concentration. In addition, trials with very fast RTs (< 250 ms) and very slow RTs (> 2000 ms) are excluded. Two participants were excluded because they did not respond properly to the task. Similar criteria were applied by Haaf and Rouder (2019). This results in 52 participants and a total of 17,031 observations. In the following two sections we will describe the model fitting and parameter estimation procedure for the normal and log-normal models, respectively.

### Normal model

#### Model fitting

The model is fitted using the packages *rstan* and *brms*. We used different settings for *rstan* and *brms* due to the difference between the models for these packages. Although we tried to make the models as similar as possible, the models are not identical. This is partly due to differences in prior specification (i.e., specifying prior on the variances versus standard deviations). For *rstan*, four chains are run with 4000 iterations per chain, of which 1000 serve as warm-up. This results in a total of 12,000 samples from the joint posterior distribution. For *brms*, four chains are run with 6000 iterations per chain, of which 1000 serve as warm-up. This results in a total of 20,000 draws from the joint posterior distribution.

#### Model diagnostics

Before we can interpret the results, we have to evaluate whether they are reliable. Specifically, we have to check whether the sampling chains of the parameters have converged, meaning that a stationary posterior distribution has been reached (Vehtari et al., 2021). If convergence is not achieved, the posterior estimates may change substantially if the sampling algorithm was run with more iterations or different starting values. The most common convergence measures are trace plots and the $\hat{R}$ statistic. We will also discuss an additional check provided by *rstan* and *brms*, namely, the number of effective samples. For a critical evaluation of currently used convergence statistics, we recommend that the interested reader consult Vehtari et al. (2021).

##### Trace plot

Trace plots show the sampled parameter value at each iteration. If the distributions have converged, the plot should look like a hairy caterpillar (Lee & Wagenmakers, 2013, p. 99): the iterations move up and down, but have much overlap in the middle. If multiple chains are used, iterations are shown per chain. In this case, the chains should overlap. If the posterior distribution did not converge, one chain could, for instance, be found on top of the figure while all other chains are on the bottom (for an illustration see Vehtari et al., 2021). Or, perhaps the first half of the chain is on the top of the graph and the second half on the bottom. The trace plot does not include burn-in samples, since these will not be used for parameter estimation.

**Fig. 9:**
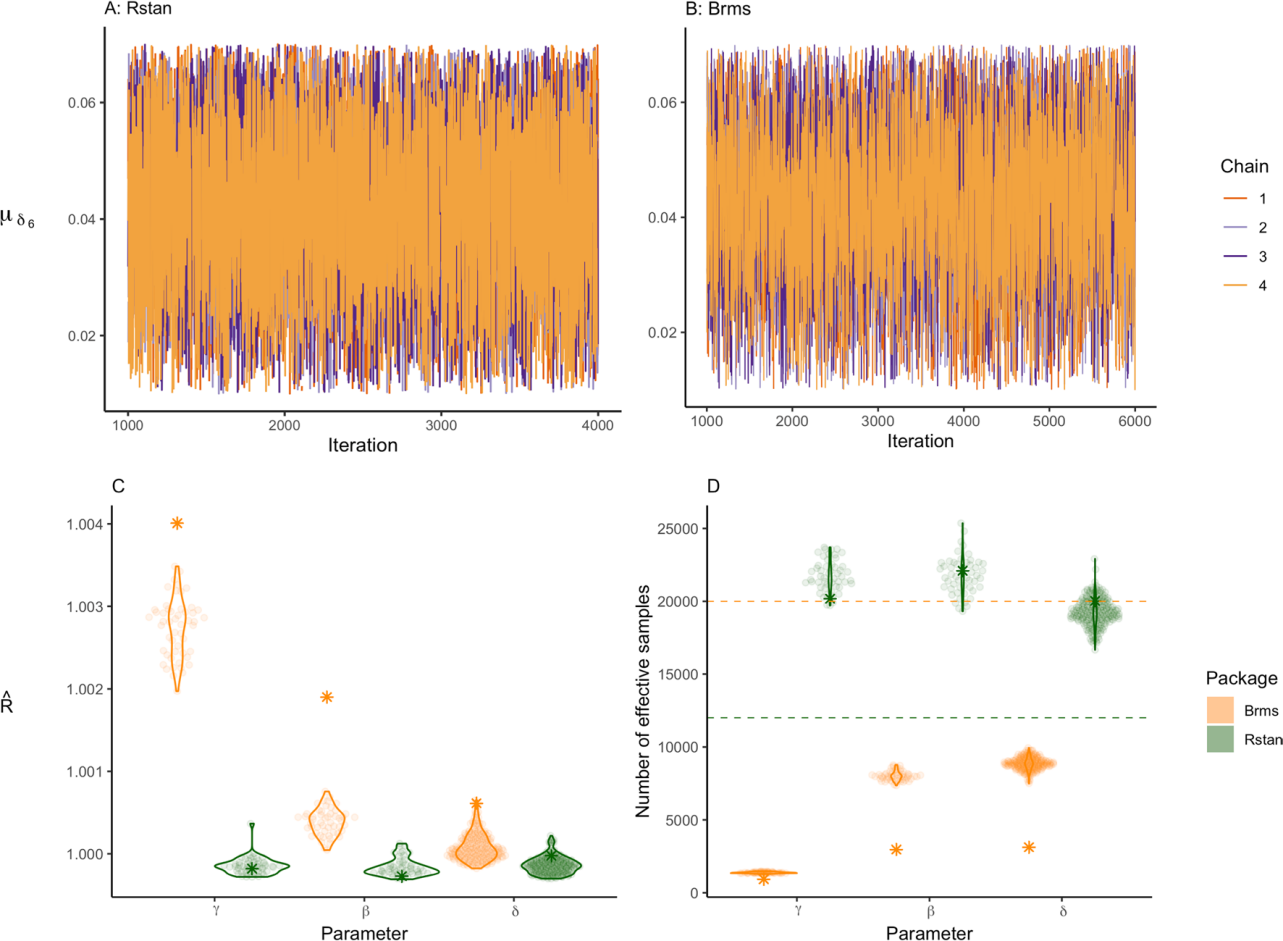
Panels A and **B** show the trace plot of the digit parameter ${\mu}_{\delta_6}$ for both packages. Panel **C** shows the estimated $\hat{R}$ for all individual effects per parameter group. The stars represent the $\hat{R}$ of the general effects. Panel **D** illustrates the variation in the estimated number of effective samples per parameter group. The dashed lines represent the total number of iterations for each package. The stars represent the number of effective samples of the general effects

When inspecting the trace plots of the symbolic distance effect parameters in the normal model, the posterior distributions of the parameters appear to have converged as the iterations come together and look similar to a caterpillar. The trace plot of one of the parameters, *δ*_6, *i*_, is shown in panel A for *rstan* and panel B for *brms* of Fig. 9. The trace plot in panel B shows less overlap in the middle, indicating that *brms* had a bit more dependence within chains. This might be a sign of slight issues, but not enough to be too concerned. Trace plots for other parameters can be found in Online Supplement G.

$$\hat{\boldsymbol{R}}$$

Next to a visual inspection of convergence, a numerical inspection is usually performed as well. A common approach is to check $\hat{R}$, also called the Gelman–Rubin diagnostic (Gelman & Rubin, 1992). $\hat{R}$ is the ratio of between-chain variance and within-chain variance (Sorensen & Vasishth, 2015). If the chains diverge, the between-chain variance will be higher than the within-chain variance, resulting in an $\hat{R}$ greater than 1. This indicates that the chains have not converged. The currently used criteria are that $\hat{R}$ should not exceed 1.01 (Vehtari et al., 2021). There are different types of $\hat{R}$, such as the split- and rank-based $\hat{R}$ (Gelman et al., 2013; Vehtari et al., 2021). Even though researchers tend to trust convergence statistics more than visual inspection, these statistics are also not foolproof. For instance, in hierarchical modeling, with many parameters, it is more likely that parameters will exceed the threshold while there are no issues with the parameter estimation. It is, therefore, advisable to evaluate parameters carefully. The development of better convergence statistics is a current topic in Bayesian analysis methods.

The $\hat{R}$ values for all individual and general symbolic distance effect parameters computed by* rstan* and *brms* are shown in panel C of Fig. 9. The figure shows that $\hat{R}$s are not substantially greater than 1.01. Therefore, it seems that the distributions have converged.

##### Number of effective samples

Another popular convergence statistic is the number of effective samples (ESS). This statistic is concerned with the dependence of posterior samples. In MCMC algorithms, the samples are to some degree dependent on one another: the parameter values at iteration *i* are similar to the parameter values at iteration *i − 1*. The number of effective samples represents the estimated total number of *independent* draws from the posterior for every model parameter (Stan Development Team, 2018a, sec. 15.4). If the number of effective samples is low, this indicates a high dependence of iterations in the sampling procedure. Therefore, the number of effective samples can be seen as a measure of the amount of new information about the posterior distribution that is provided by the total number of samples drawn from the posterior. A common rule for interpretation is that the number of effective samples should equal at least 100 per chain (Vehtari et al., 2021). In the case of four chains, this would mean a value of 400 or higher—the higher the value the better. To get a better understanding of convergence, Vehtari and colleagues (2021) recommended evaluating different quantities of the posterior distribution instead, using so-called bulk- and tail-ESS. Bulk-ESS evaluates the center of the posterior distribution, while tail-ESS evaluates the tails of the posterior distribution. For this tutorial, we focus on the basic calculation of the number of effective samples suggested by Gelman et al. (2013) and improved by Vehtari et al. (2021).

For the symbolic distance effect, the estimated number of effective samples is provided in panel D of Fig. 9. For the package *rstan* the number is generally high, much higher than the actual number of samples depicted by the green dashed line. The *brms* package shows much more variation. The lowest effective sample sizes can be found for the intercept parameters. Table 1 also displays the number of effective samples for the general effects parameters in the *rstan* model. If the number of effective samples is too low, *brms* and *rstan* will return a warning. Here, this was not case, and all other diagnostics were good, so we continue with the interpretation of the estimates. The difference between the model diagnostics of* rstan* and *brms* can be partly explained by the difference in model parametrization, such as the difference in the prior specification (i.e., specifying priors on variances versus priors on standard deviations). We equated the models where sensible, which resulted in models that are fairly comparable.

**Table 1:** Posterior variance, lower and upper bound of the 95% credible interval, number of effective samples, and $\hat{R}$ of the variance parameters as estimated by r*stan*

Parameters	Mean	Lower bound	Upper bound	*n*_*eff*_	$\hat{R}$
${\sigma}_{\gamma}^2$	0.034	0.024	0.049	17,556.041	1.000
${\sigma}_{\beta}^2$	0.020	0.014	0.029	20,115.358	1.000
${\sigma}_{\delta_7}^2$	0.020	0.014	0.029	20,340.216	1.000
${\sigma}_{\delta_6}^2$	0.020	0.014	0.029	19,716.061	1.000
${\sigma}_{\delta_4}^2$	0.020	0.014	0.029	18,989.012	1.000
${\sigma}_{\delta_3}^2$	0.020	0.014	0.029	18,357.620	1.000

#### Estimation results

Considering acceptable to good convergence diagnostics, we can now interpret the estimation results. First, we present the general effects, then we visualize the individual variability of these effects.

##### General effects

For the symbolic distance effect, there are two main questions we want to answer. First, we want to know whether there is a digit effect and, second, we want to know whether there is a side effect. Therefore, the key model parameters for general effects are *μ*_*β*_, ${\mu}_{\delta_3}$, ${\mu}_{\delta_4}$, ${\mu}_{\delta_6}$, and ${\mu}_{\delta_7}$. In the following section, we will focus our reporting on these parameters.

**Fig. 10:**
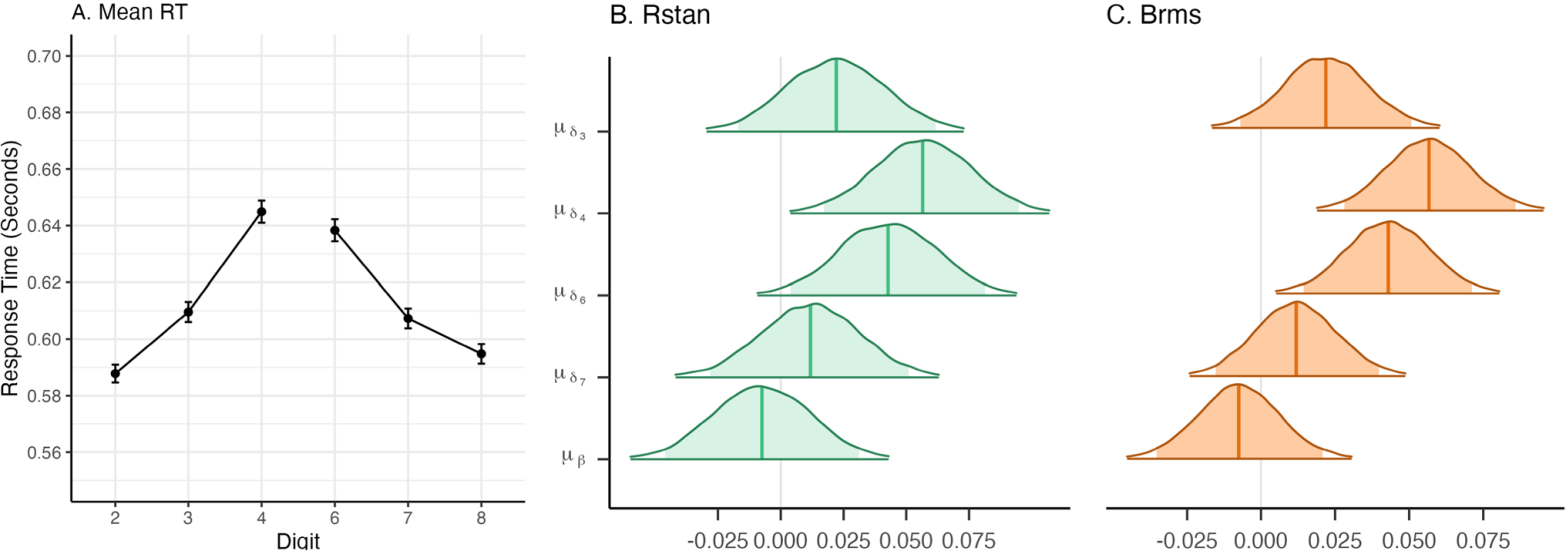
Panel **A** shows the empirical mean RTs per digit. The standard error is represented by bars around the dots. Panels **B** and **C** show the posterior distributions for the general effects estimated by the two packages. The middle line within the distributions represents the posterior mean. The shaded area within the distributions represents 95% credible intervals.

The posterior distributions for the focal parameters in the normal model are presented in Fig. 10. The posterior means estimated by *rstan* and *brms* can be found in Online Supplement H. The credible interval displayed in the figure is the Bayesian version of a confidence interval and provides a measure of uncertainty about the parameter value (Wagenmakers et al., 2018). In contrast to the confidence interval, the credible interval provides the probability that a parameter lies within a certain range. However, the credible interval does not provide evidence to conclude whether an effect is zero (Wagenmakers et al., 2020).

The parameter estimates produced by the two packages look very similar. The *brms* estimates seem to be slightly higher with slightly narrower posterior distributions compared to *rstan*. All parameters are presented on a scale of seconds. As expected, the effects are small. For instance, ${\mu}_{\delta_7}$ has a posterior mean of 0.012, corresponding to a 12-millisecond effect.

For the digit effects, positive parameter estimates indicate that the response is slower for the non-baseline digits, that is, digits that are closer to 5. The parameters ${\mu}_{\delta_6}$ and ${\mu}_{\delta_4}$ are distributed around higher values. Note that this result is consistent with the symbolic distance hypothesis that the digits 4 and 6 have the highest response times because they are closest to 5, the comparison value. The posterior distribution of the side effect *μ*_*β*_ is centered around zero with a posterior mean of −0.007 and a 95% credible interval ranging from −0.046 to 0.031. Note that the credible interval overlaps with zero. Commonly, this is misinterpreted as an absence of an effect. However, credible intervals should only be used for parameter estimation. In cases where the credible overlaps with zero, the effect is likely small, but can be nonzero.

##### Individual effects

Thanks to the hierarchical structure of the model, we can investigate whether the general effects hold for all the individuals by inspecting the individual variation and the individual parameter estimates. The individual variation is represented by the variance or standard deviation parameters in the model. The summaries of the posterior distributions of the variance parameters as estimated by *rstan* are shown in Table 1. The estimates for the standard deviations by *brms* are smaller than those for *rstan* and can be found in Online Supplements D and H.

Posterior means of the variances for the digit and side effects are all very close to 0.020, which corresponds to a standard deviation of 0.141 seconds, that is, 141 milliseconds. Additionally, the 95% credible intervals do not contain zero. If we consider the effect of digit 7 with an 12-millisecond general effect, this is a substantial amount of individual variation. Therefore, we conclude that there are individual differences in the symbolic distance effect.

**Fig. 11:**
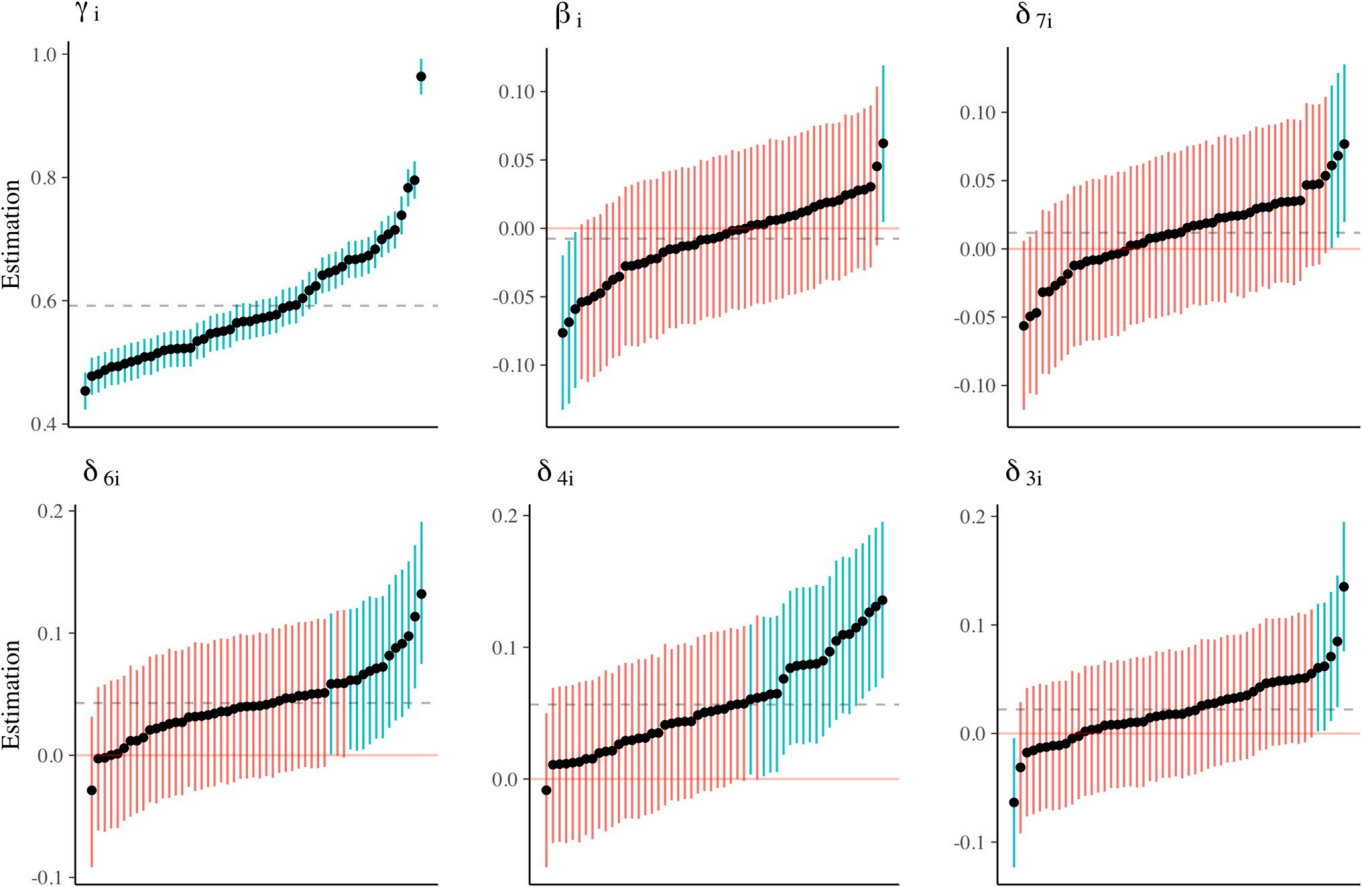
The posterior means for individual effect parameters with 95% credible intervals as estimated by *rstan*, shown in increasing order. The dashed line represents the general posterior mean. Pink intervals contain zero; blue intervals do not contain zero

Figure 11 shows the posterior means and 95% credible intervals for the individual effects of all 52 participants. Estimates are shown in increasing order, from lowest to highest. Pink intervals indicate that the 95% credible interval contains zero, while blue intervals are either entirely above or below zero. A corresponding figure with individual effect estimates for *brms* can be found in Online Supplement D.

The largest variation across participants can be seen for the intercept parameter. This means that participants vary considerably in their baseline response times. There is also considerable individual variation in the digit effects (*δ*s). However, most of the intervals contain zero, which implies that the effects might be small. The variability is most pronounced for the effect parameters of the digits close to 5. For these digits, there are also more intervals that do not contain zero, indicating that an effect might be present on the subject level. The posterior mean of the individual side effect (*β*_*i*_) varies between −0.05 and 0.05. Again, all but four 95% credible intervals include zero, indicating uncertainty about the existence of an effect for anyone. The credible intervals of the side effect and digit effects *δ*_7_ and *δ*_3_ indicate that different subjects may show opposite effects (Haaf & Rouder, 2019).

**Fig. 12:**
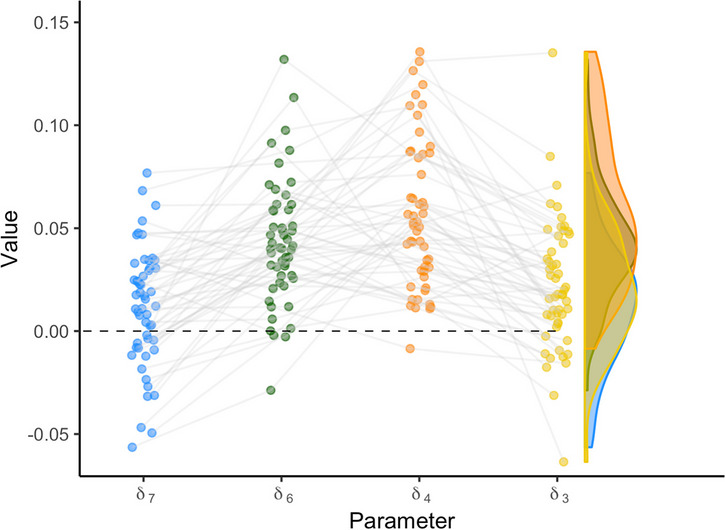
Model estimates for digit effect parameters in *rstan*. The points represent the mean parameter estimates for each individual. The violin plots on the right side show the variance in the individual parameter estimates.

The individual variation can also be visualized differently, for example by connecting individual estimates for each parameter. This type of variation can reveal correlations between individual parameter estimates; for example, participants scoring high on one digit parameter may also score high on another digit parameter. Figure 12 shows the relationship between individuals’ digit effects.^7^ The posterior means of individual effects are represented by the dots. The right side of each plot represents the distribution of the individual estimates. Note that this distribution is not the posterior distribution of the general effect. It rather corresponds to the left side of Fig. 7.

### Log-normal model

#### Model estimation

The model is fitted using the packages *rstan* and *brms*. For *rstan*, we use the priors as specified in Eq. (8). For *brms*, we use the priors in Eq. (9). The rest of the settings are equivalent for both packages. We ran four chains with 4000 iterations per chain, including 1000 warm-up iterations. This results in a total of 12,000 samples from the posterior distribution.

#### Model diagnostics

**Fig. 13:**
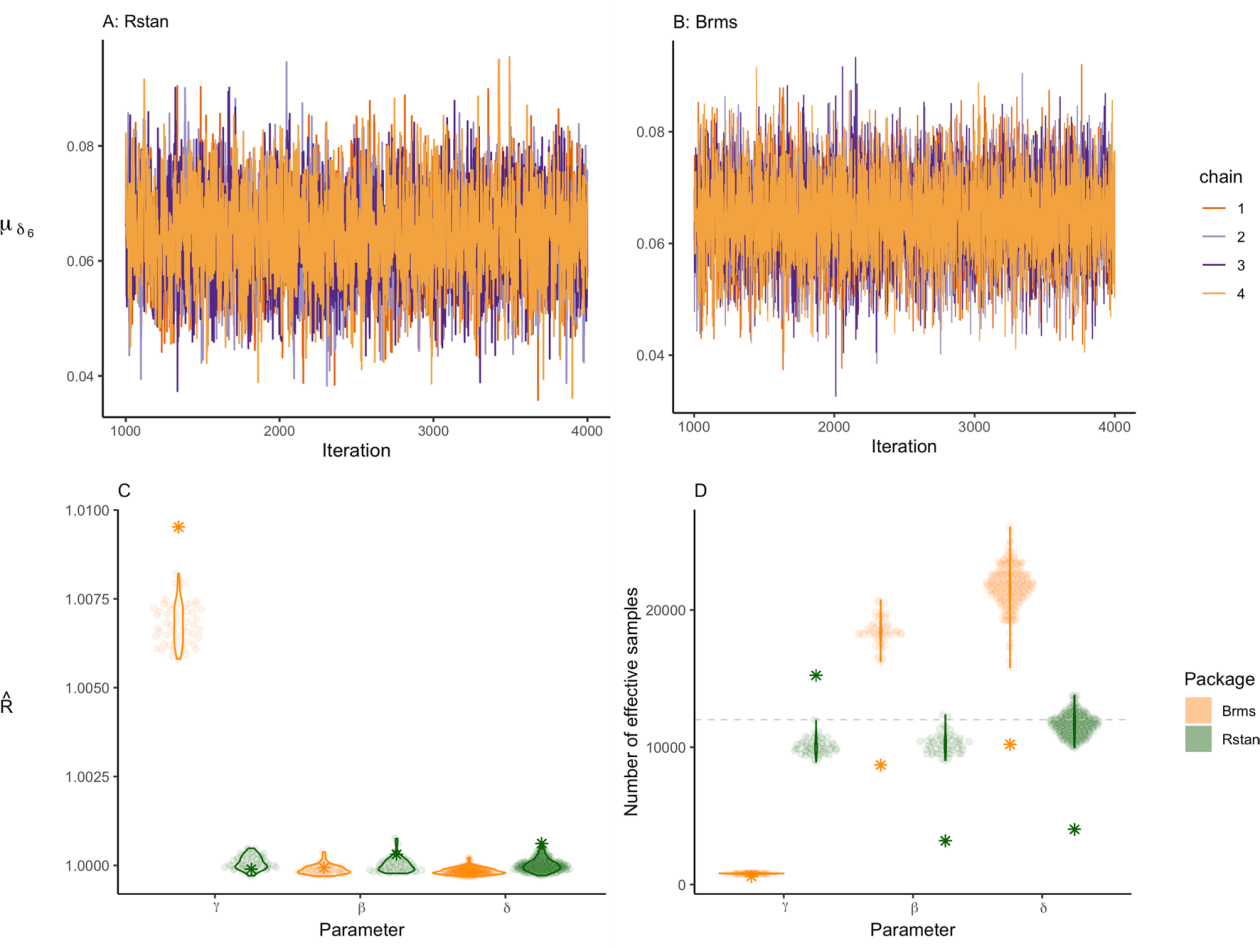
Panel **A** and **B** show the trace plot of the digit parameter ${\upmu}_{\updelta_6}$in the log-normal model for both packages. Panel **C** shows the estimated $\hat{R}$ for all individual effects per parameter group. The stars represent the $\hat{R}$ of the general effects. Panel **D** illustrates the variation in the estimated number of effective samples per parameter group. The dashed lines represent the total number of iterations for each package. The stars represent the number of effective samples of the general effects.

Before interpreting the results, we have to check whether the posterior distributions of the parameters have converged. The trace plot of ${\mu}_{\delta_6}$ is shown in panels A and B of Fig. 13 for *rstan* and *brms*, respectively. Trace plots for all other parameters can be found in Online Supplement G. All plots look like a hairy caterpillar. Therefore, they indicate that the posterior distributions of the parameters have converged. $\hat{R}$ values shown in Fig. 13C for all parameters are close to 1, and in most cases, the number of effective samples shown in Fig. 13D exceeds the total number of iterations. The number of effective samples for the general effects is comparatively low but still sufficient. Considering these in combination with the other diagnostics, we conclude that the posterior distributions of the parameters have converged and we proceed with the interpretation of the results.

#### Results

##### General effects

**Fig. 14:**
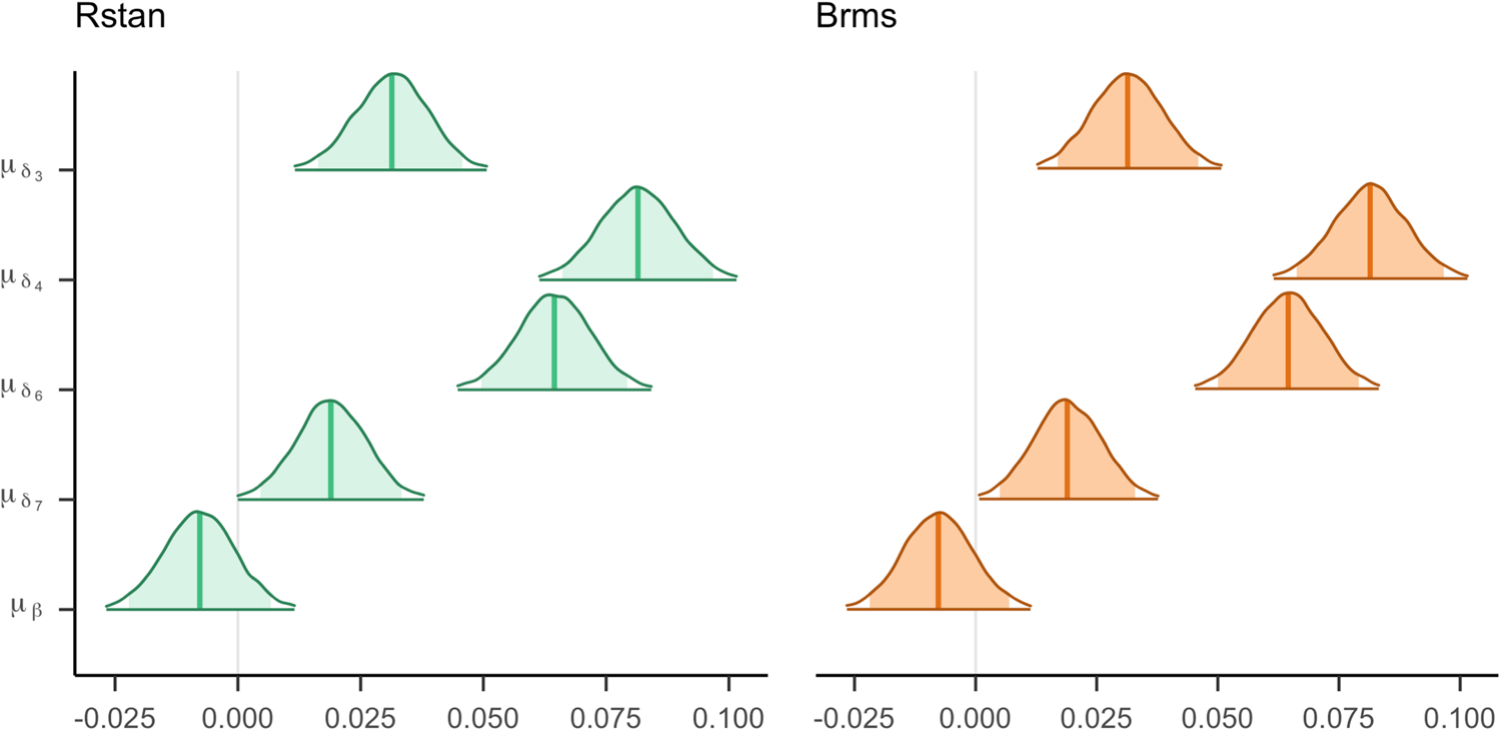
The posterior distributions for the general effects in the log-normal model estimated by the two packages. The middle line within the distributions represents the posterior mean. The shaded area within the distributions represent 95% credible intervals

We inspect the posterior distributions for the general effect parameters presented in Fig. 14.

We note that the values of the parameter estimates for the log-normal model cannot be interpreted in the same way as in the normal model. This is because the mean parameter of the log-normal distribution does not correspond to its expected value. To better understand the size of an effect that corresponds to the parameter values, some calculations are required. For instance, if we would like to know the median difference in response times for digits 3 and 2, we calculate the difference between the exponential of the RT estimate for the condition digit = 3 and the exponential of the RT estimate for the condition digit = 2:

12 $${\displaystyle \begin{array}{cl}& {e}^{\left({\mu}_{\gamma }+{x}_j{\mu}_{\beta }+{w}_j{\mu}_{\delta_3}\right)}-{e}^{\left({\mu}_{\gamma }+{x}_j{\mu}_{\beta}\right)}\\ {}& ={e}^{\left(-.56+.5\times \left(-.01\right)+1\times 0.03\right)}-{e}^{\left(-.56+.5\times \left(-.01\right)\right)}\\ {}& =0.017\end{array}}$$

This value corresponds to the median estimate of ${\mu}_{\delta_3}$ from the normal model. Calculating the mean estimate of ${\mu}_{\delta_3}$ would be similar, but more complicated, as the mean of a log-normal distribution also depends on its variance. This approach can also be used iteratively. This means that the approach above is applied to every iteration in the chain. This results in a posterior distribution of the effect quantifying the posterior uncertainty.

One property of these parameter estimates we can immediately interpret is their sign (i.e., positive, negative, or zero). A positive estimate indicates that the RT increases, whereas a negative effect indicates a decrease. When inspecting the general digit effect parameters, they all appear to positively influence the RT. This is in line with our expectations, since the baseline RT in the model is set equal to the RT for the digits furthest away from 5, and the symbolic distance effect postulates that RT should increase as the digits become closer to 5. The side effect is close to zero, with a posterior mean of −0.008 and a 95% credible interval from −0.022 to 0.007. This indicates that the general effect of side is small.

##### Individual effects

Next, we inspect the individual deviations from the general effects. Table 2 presents the estimates of the variance parameters by *rstan*. Results for *brms* can be found in Online Supplement H.

**Table 2:** Posterior variance, lower and upper bound of the 95% credible interval, the number of effective samples, and the $\hat{R}$ of the variance parameters as estimated by *rstan*

Parameters	Mean	Lower bound	Upper bound	*n*_*eff*_	$\hat{R}$
${\sigma}_{\gamma}^2$	0.030	0.021	0.043	15,617.383	1.000
${\sigma}_{\beta}^2$	0.001	0.001	0.002	7,815.681	1.000
${\sigma}_{\delta_7}^2$	0.001	0.001	0.002	6,805.297	1.000
${\sigma}_{\delta_6}^2$	0.001	0.001	0.002	7,325.299	1.000
${\sigma}_{\delta_4}^2$	0.001	0.001	0.002	6,237.497	1.000
${\sigma}_{\delta_3}^2$	0.001	0.001	0.002	6,841.198	1.001

The estimated parameter values for the variance terms are very small, but different from zero. This indicates that individuals may vary slightly with respect to the general effect parameters. The estimates for the standard deviations by *brms* are similar to the estimates by *rstan*, but slightly smaller. The exact estimates by *brms* can be found in Online Supplements F and H.

**Fig. 15:**
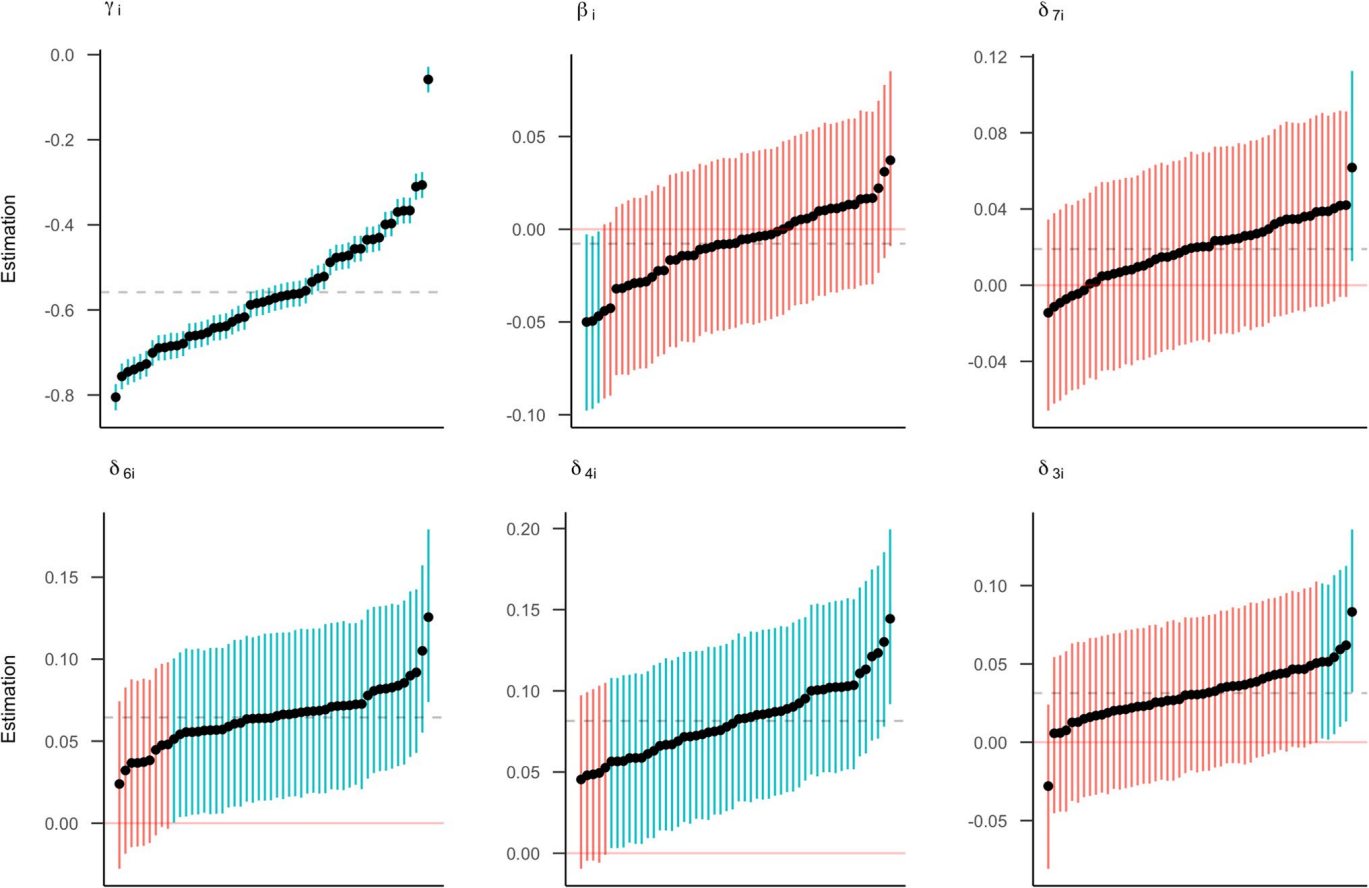
The posterior means for individual effect parameters in the log-normal model with 95% credible intervals as estimated by *rstan*, shown in increasing order. The dashed line represents the general posterior mean. Pink intervals contain zero; blue interval do not contain zero

Figure 15 shows the individual estimates for the digit and side effects. The pink lines indicate that the 95% credible interval of the individual effect contains zero. The estimated general effect is represented by the dashed line. For *β*, *δ*_7_, and *δ*_3_, most 95% credible intervals contain zero. For *δ*_6_ and *δ*_4_, most credible intervals are entirely larger than zero, indicating an increase in RT for most participants.

**Fig. 16:**
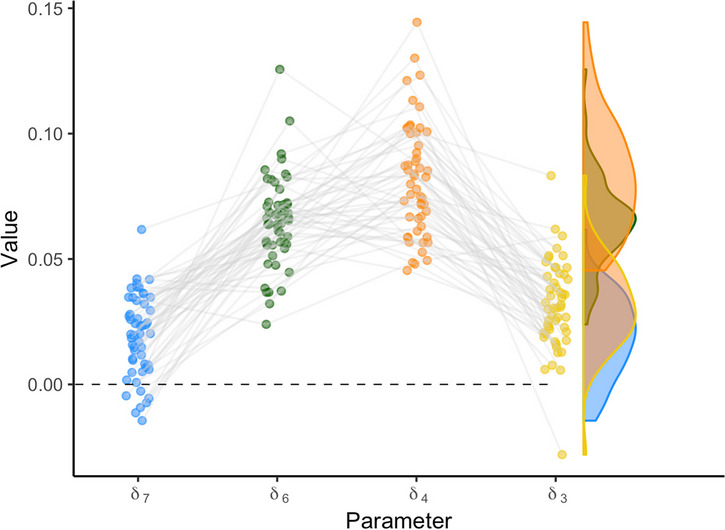
Model estimates for digit effect parameters in the log-normal model in *rstan*. The points represent the mean parameter estimates for each individual. The violin plots on the right side show the variance in the individual parameter estimates

We can further inspect the individual estimates for the digit effects in Fig. 16. The figure shows the individual estimates for the digit effects with the distribution of the variability in the individual estimates (not the posterior distribution of the general effect). The figure illustrates that there is considerable variance in the point estimates of the individual digit effects. However, compared to the normal model there is considerably more hierarchical shrinkage. This means that in the log-normal model, the individual effects are corrected more towards the group mean, compared to the normal model. Or, the group mean in the log-normal model is not affected as much by individuals showing a divergent trend compared to the normal model. Whether we should prefer a model with more shrinkage depends on the theoretical considerations. If more individual variation is expected, less shrinkage would be preferred. However, the advantage of more shrinkage is that outliers do not influence the estimation of the effect as much.

## Model comparison

The symbolic distance effect postulates that response times (RTs) are slower when digits are closer to 5 (digit effects), and that RTs may be influenced by whether the target digit is smaller or larger than 5 (side effect). Using Bayesian estimation, we are not able to directly test these hypotheses. Credible intervals often contain zero, but they always also contain many other values that have a chance of being the “true” parameter value, making them unsuitable for testing hypotheses. Therefore, our goal for the following section is to set up a Bayesian hypothesis test for the parameters of interest in our model. Specifically, we will achieve this by comparing the predictive accuracy of a null model where we set one or some parameters to zero, and effects models where these parameters are unconstrained. This approach is called Bayes factor model comparison.

### Bayes factor

Rather than submerging us in technical details or providing a formal justification of the Bayesian approach, we provide an intuition of Bayesian model comparison. A formal justification of Bayes factors is provided by Jeffreys (1961), Kass and Raftery (1995), and Rouder and Morey (2018). The Bayes factor (BF) is a measure of how well one model predicts the data compared to another.

Bayesian analysis allows for predictions of data from any model because models are fully specified with a probability distribution for the data and priors for all parameters. Let the vector of all parameters of a model be **θ**. With the prior distributions of these parameters, *f*(**θ**), the prediction for data from the model can be computed by integrating over all parameters:

13 $$p\left(\textbf{Y}|\mathcal{M}\right)={\int}_{\boldsymbol{\Theta}}p\left(\textbf{Y}|\mathcal{M},\boldsymbol{\uptheta} \right)f\left(\boldsymbol{\uptheta} \right)d\boldsymbol{\uptheta} .$$

Note that ∫_**Θ**_ refers to a multidimensional integral over the entire parameter space. As our models consist of many parameters, computing these predictions for any one model becomes somewhat difficult. Once the integral is computed, the resulting probability distribution of the data, $p\left(\textbf{Y}|\mathcal{M}\right),$ is no longer a function of the model parameters. This marginal probability distribution is therefore also referred to as the marginal likelihood. Before observing the data, the marginal likelihood serves as prediction for possible data; once the data are observed, the function tells us how well these specific observations were predicted by the model. The Bayes factor then is the ratio of marginal likelihoods of two models, or the relative predictive accuracy of ${\mathcal{M}}_1$ over ${\mathcal{M}}_2$:

14 $${\mathrm{BF}}_{1,2}=\frac{P\left(\textbf{Y}|{\mathcal{M}}_1\right)}{P\left(\textbf{Y}|{\mathcal{M}}_2\right)}.$$

There is a second popular interpretation of the Bayes factor as the relative evidence of two competing models. One of the key insights from Bayes’ rule is that these two things, the relative predictive accuracy of two models for the data and the relative evidence from the data for the two models, are one and the same (Rouder & Morey, 2018).

Bayes factors have a few very convenient characteristics. One of them is that Bayes factors are transitive; that is, evidence for the full model over the null model can be obtained by flipping the numerator and denominator in the equation above. For a full overview of the advantages of Bayesian inference with Bayes factor, see Wagenmakers et al. (2018).

Imagine that BF_1, 2_ = 100. This means that the data are 100 times as likely under the normal model as under the log-normal model, clear evidence in favor of the normal model. But how do we know whether a Bayes factor of 100-to-1, or 10-to-1 or 3-to-1 is big enough? As a reminder, Bayes factors are the relative predictive accuracy of two models for the observed data. Therefore, Bayes factors are odds or ratios. Odds themselves are directly interpretable without the need for decisions or cutoffs. For example, if a presidential candidate is favored 10-to-1 over another one, then these are just the odds, and it is beside the point whether these are large or not. According to this line of thought, Rouder et al. (2018) argue that researchers should take a similar approach when interpreting and reporting Bayes factors. While there exist rules of thumb to categorize Bayes factors verbally, we suggest researchers use their research context to form substantive conclusions based on Bayes factors, not arbitrary rules of thumb.

As in the case of the posterior distribution, Bayes factors can rarely be computed analytically. The difficulty in computing Bayes factors is one of the major drawbacks of Bayesian inference. However, several algorithms are available to estimate the Bayes factor from posterior samples. In the following section we will present two methods to obtain Bayes factors for the symbolic distance effect. The first method that we discuss, the Savage–Dickey density ratio, tests the general effect while disregarding individual variation (similar to type III sums of squares in ANOVA models). This means that the compared models differ in only a single parameter, that is, the general effect parameter, and individual effects are present in both models. The Savage–Dickey density ratio is computationally efficient, but can only be used for tests of a single parameter value as described earlier. This is why we additionally introduce a second approach, bridge sampling, that is less computationally efficient but more versatile. In this manuscript, we will use bridge sampling to test the general effects and individual variation together, as well as to compare the predictive accuracy of the normal and log-normal model. There have been discussions on which method should be preferred, without consensus (van Doorn et al., 2021, 2023; Rouder et al., 2023; Singmann et al., 2023). Therefore, we illustrate the use of the two approaches and discuss their potential.

#### Savage–Dickey density ratio

One method for computing Bayes factors is the Savage–Dickey density ratio (SDD-ratio; Dickey & Lientz, 1970; Morey et al., 2011; Wagenmakers et al., 2010). This approach can be readily used to estimate the Bayes factor between two nested models that differ in only one parameter. A prime example for such a case is comparing an effect model with a null model. For example, we may compare a model with an overall side effect (effect model) to a model with no overall side effect (null model). In this case, the Bayes factor in favor of the side effect can be approximated by the ratio of the posterior density of the side effect and the prior density of the side effect at the point zero. More generally, the Savage Dickey approach works whenever one model fixes a parameter to a constant and another model assigns a prior distribution to the same parameter.

**Fig. 17:**
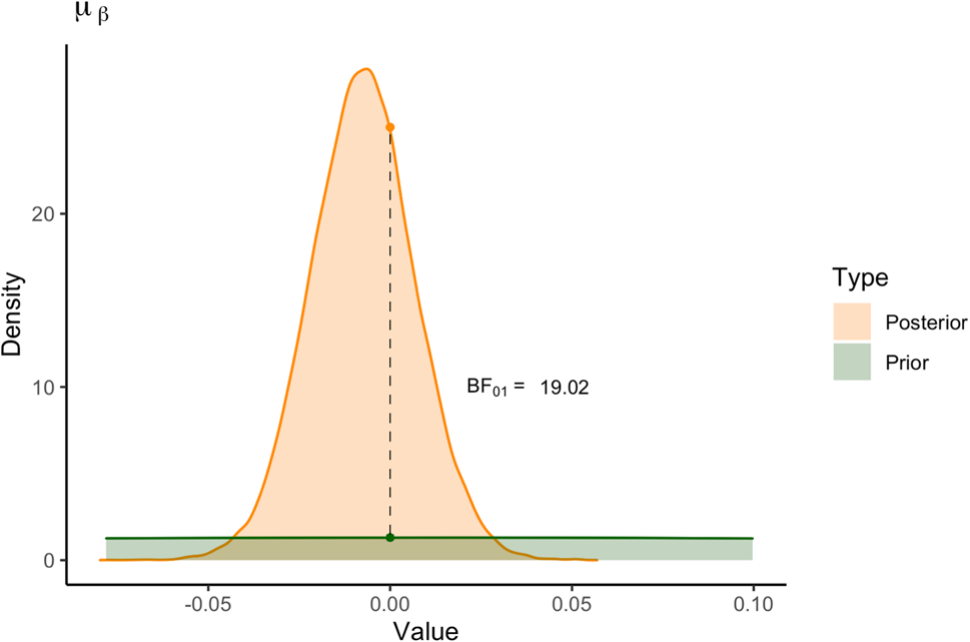
The prior and posterior distribution of the side parameter estimated by the *brms* package. The dots represent the height of the distributions at zero. The ratio of these dots indicates the Savage–Dickey density ratio. The Bayes factor represents the evidence for the hypothesis that the effect equals zero. A Bayes factor above 1 indicates that the evidence favors the null hypothesis.

Figure 17 shows a visualization of the SDD-ratio for the side parameter in the normal model of the symbolic distance effect. The posterior distribution is depicted in orange, the prior in green. The Bayes factor can be computed as a ratio of the posterior and prior density at the parameter value of zero, as depicted by the dashed line connecting the two points on the density functions.

With *rstan*, the SDD-ratio can be computed by fitting a density function to the posterior distribution obtained from the MCMC samples, and comparing the value of this density function at *μ*_*β*_ = 0 to the value of the prior distribution at *μ*_*β*_ = 0. This is illustrated in the code block below. First, we extract the posterior samples for the parameter *μ*_*β*_, the general effect of side. We apply the *logspline* function (Kooperberg, 2019) to the samples to obtain an estimate of the log-density of the posterior distribution. The density at zero is calculated with the *dlogspline* function. Finally, we compare the estimated density of the posterior to the density of the prior. This process is explained in further detail by van Ravenzwaaij and Etz (2021).

 In *brms*, the SDD-ratio can be computed using the built-in hypothesis function, as shown in the code snippet below. The resulting Bayes factor, also depicted in Fig. 17, is *BF*_01_ = 18.28, indicating evidence for the null model and against a side effect.

 The Savage–Dickey approach has a number of drawbacks (see, for example, Heck, 2019). First, one issue is the quality of approximation for the SSD-ratio. If either the prior or, even more problematically, the posterior has a very low density at the test value (in this case zero), it becomes difficult to estimate the SSD-ratio from the samples. This issue often occurs when the effect is large, moving the posterior distribution away from zero. In many cases there might be no samples around zero, which means that the density estimate will be very inaccurate. Therefore, we would recommend using the SSD-ratio only if you are confident that enough posterior samples are available from the tails of the distribution. One way of ensuring that is to drastically increase the number of drawn samples.

Second, the Savage–Dickey approach only applies for a limited set of to-be-compared models. In the example used here, we compared a model where the overall side effect is allowed to vary with a model where it is zero. Both models, however, still allow for individual variability in the side effect. For the null model, individual variability implies that some individuals are expected to have an effect favoring larger numbers and other individuals are expected to have an effect favoring smaller numbers, but, on average, both groups perfectly balance out at zero. To us, this model seems nonsensical and, more importantly, it is not what researchers have in mind if they want to test a model without side effect. We would prefer to use a null model where none of the participants have a side effect. However, the Savage–Dickey density ratio is not available for such a model compared to the effects model.

#### Bridge sampling

Bridge sampling offers a more flexible way to obtain Bayes factors (Kass & Raftery, 1995). It allows the computation of Bayes factors for comparing models that differ in more than one parameter. Specifically, bridge sampling is a sampling-based algorithmic method to obtain the marginal likelihood of the data under a given model from the posterior samples (Gronau et al., 2017; Meng & Wong, 1996).

Using bridge sampling we are able to estimate Bayes factors between any models of interest for the classification task data. We identify four models of interest:

1. Full model: This is the model we have been using throughout this paper. It states that there is an effect of side and an effect of digits for all participants.
2. Side model: This model specifies that participants show a side effect but no digit effect. All digit parameters *δ*_3_ to *δ*_7_ are set to zero.
3. Digit model: This model states that there are no side effects but that participants exhibit digits effects. All side parameters *β* are set to zero.
4. Null model: In this model, there is no side or digit effect on the RT. The digit parameters *δ*_3_ to *δ*_7_ and side parameters *β* are set to zero.

Note that models 2–4 are nested in the most complex full model, and the null model is nested in models 2 and 3, but models 2 and 3 are not nested. Additionally, the models differ by several parameters. For example, compared to the full model, the digit model restricts the side parameter for all participants to zero. Using bridge sampling, we can estimate the marginal likelihood for each model separately, and then compute Bayes factors as their ratios [Eq. (14)].

To obtain the marginal likelihood for a specific model in* rstan* with bridge sampling (Gronau et al., 2017), the *bridgesampling* package can be used (Gronau et al., 2020). To obtain the Bayes factor comparing the side model and the full model, we first have to fit both models. Then, we use the *bridge_sampler* function from the *bridgesampling* package to obtain the marginal likelihood of the data under each of the models. Finally, the models are compared using the *bf* function from the *bridgesampling* package.

 In the *brms* package, Bayes factors based on bridge sampling can be obtained with the function *bayes_factor* as shown in the code snippet below. The function takes two fitted models as input. Therefore, both models first have to be fitted as explained in the “Model estimation” section of this manuscript.

 Even though bridge sampling is a flexible method for obtaining Bayes factors, it is still an estimation method based on posterior samples. Therefore, as with any Bayes factor estimation method, we advise taking two precautions with bridge sampling. First, it is always wise to increase the number of iterations when performing model comparison. This is because convergence of estimation of posterior distributions and convergence of estimation of the marginal likelihood can differ. Therefore, a rule of thumb is to use ten times the number of iterations that we would usually use for estimation. The second precaution is to conduct a stability analysis. Both the posterior samples from the model estimation procedure and the estimate of the marginal likelihood from the bridge sampling can be variable. Therefore, stability may be best assessed by repeating the entire process of fitting each model and applying the bridge sampling function several times.

**Fig. 18:**
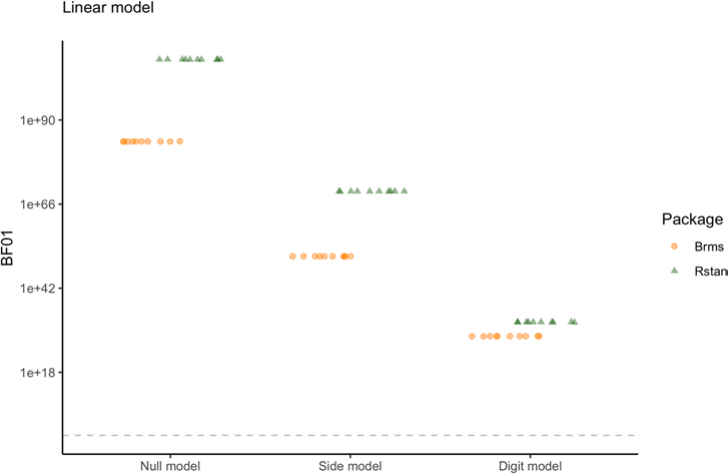
The BF estimates of the other models (0; null, side, and digit model) against the full model (1). The green triangles represent the estimates using rstan. The orange dots represent the brms estimates. The dashed line represents a BF_01_ of 1

We assess the stability of the obtained Bayes factor by repeating the estimation process ten times with *rstan* and *brms*, respectively. The results are shown in Fig. 18. The figure shows the evidence in favor of the null, digit, and side model against the full model. All Bayes factors are larger than 1. Therefore, it can be concluded that the data are more likely under less complex models. There is almost no variation in the computed Bayes factors per model, indicating that the Bayes factor estimate is stable. However, the Bayes factor estimates in *rstan* generally show stronger evidence than Bayes factor estimates based on *brms*. It is important to note that this difference is not due to the bridge sampling function (i.e., there is little to no variation in Bayes factor estimates within packages), but a result of a difference in parametrization. The models for *rstan* and *brms* are similar but not identical, partly due to the difference in prior specification (i.e., variances versus standard deviations). However, for both packages, the conclusion is the same: the data are least likely to have occurred under the full model. If the estimates are unstable, further increasing the number of posterior samples is recommended.

Finally, it is time to answer our research question: Is there a symbolic distance effect? When considering all Bayes factor estimates from Figs. 17 and 18, the null model (BF_*max*_ = 232 × 10^105^ for *rstan*) performs best compared to the side model (BF_*max*_ = 458 × 10^68^ for *rstan*) and the digit model (BF_*max*_ = 269 × 10^31^ for *rstan*). Therefore, the data from Rouder et al. (2005) show evidence against the symbolic distance effect.

### Sensitivity analysis

The full model including the side and digit effects performs worst of all models in the Bayes factor model comparison. However, we did find nonzero digit effects in the model estimation. How is this possible?

A possible explanation is a lack of individual variability in the digit and side effects. The full model assumes that participants vary in the digit and side effect, and we specified our expectations about this size of the variability in the prior. However, if there is a lack of individual variability in the effects, the full model is punished for the complexity added by the individual variation (illustrated by Rouder & Haaf, 2021). To investigate this idea, we can reformulate the prior distributions on the variance parameters to indicate a smaller individual variation or, even more extreme, remove these individual effects from the model entirely. Then we can calculate the posterior model probabilities for all specified models. Typically, sensitivity analyses assess the sensitivity of the Bayes factor by the width of the prior on effect sizes (e.g., van Doorn et al., 2021). However, in our case the prior distributions on the variance parameters are key to understanding sensitivity.

Specifically, we will compare models from three scenarios: scenario 1 specifies large individual differences equivalent to Eq. (6). For the models in scenario 2, we adjust the priors on ${\sigma}_{\beta}^2$ and ${\sigma}_{\delta}^2$ in the following way:

15 $${\displaystyle \begin{array}{rr}{\sigma}_{\beta}^2& \sim \mathrm{Inverse}-\mathrm{Gamma}\left(3,0.05\right),\\ {}{\sigma}_{\delta}^2& \sim \mathrm{Inverse}-\mathrm{Gamma}\left(3,0.05\right).\end{array}}$$

The smaller value for the scale parameter of the inverse gamma distribution (i.e., 0.05 instead of 0.5) results in a shift of the peak of the prior towards smaller values for the individual variance. This means that the variation of the individual effects is expected to be much smaller than before.

In scenario 3, we remove individual variability from the side, digit, and full model. This results in the following full model, from which the side and digit model follow logically:

16 $${Y}_{ijk}\sim \mathrm{Normal}\left({\gamma}_i+{x}_j{\mu}_{\beta }+{u}_j{\mu}_{\delta 7}+{v}_j{\mu}_{\delta 6}+{w}_j{\mu}_{\delta 4}+{z}_j{\mu}_{\delta 3},{\sigma}^2\right).$$

Note that the null model is the same in all three scenarios.

To obtain the posterior model probability for each model in every scenario, we first compute the Bayes factor of every model against the null model. This includes the trivial Bayes factor of the null model against the null model. This setup results in 10 models and, therefore, 10 Bayes factors. We assume that all models are equally likely, resulting in a prior model probability for each model of 1/10. Next, we calculate the sum of all these Bayes factors and divide each Bayes factor by this sum. This results in the posterior model probabilities shown in Table 3.^8^ All posterior model probabilities from the table and the posterior probability of the null model (see table note) sum to 1.

**Table 3:** Posterior model probabilities normal model

	Scenario 1	Scenario 2	Scenario 3
Digit model	0.87 × 10^−126^	0.29 × 10^−46^	0.95
Side model	0.20 × 10^−88^	0.18 × 10^−66^	0.77 × 10^−53^
Full model	0.43 × 10^−158^	0.47 × 10^−61^	0.05

*Note.* Scenario 1: large individual differences. Scenario 2: small individual differences. Scenario 3: no individual differences. The posterior probability of the null model is the same in every scenario and equals 0.50 × 10^−107^ .

The table shows that the digit model without individual digit effects from scenario 3 has the highest posterior model probability, followed by the full model without individual digit and side effects (scenario 3). This means that the data are most likely under these models than the other eight models included in the comparison. In addition, the models with smaller prior expectations for individual differences (scenario 2) have higher posterior model probabilities than the models in scenario 1. Thus, decreasing the variability in the size of the individual digit and side effects (scenario 2), or completely removing the variance (scenario 3), results in higher posterior model probabilities. Together with Fig. 11, showing the small differences in individual effects (also illustrated by Haaf et al., 2019), this result supports the notion that side and digit effects are small and there is a lack of individual variability.

#### Log-normal model

We can take a similar approach for the log-normal model to compare the hierarchical models on varying complexity. We evaluate their performance in two scenarios that correspond to scenario 1 and 3 for the normal model. This means that in scenario 1 for the log-normal model, we estimated a null, digit, side, and full model based on the prior specified in Eq. 8, specifying large individual differences. For scenario 2, we remove individual variability from the side, digit, and full model. This resulted in the posterior model probabilities shown in Table 4.

**Table 4:** Posterior model probabilities log-normal model

	Scenario 1	Scenario 2
Digit model	26.61 × 10^9^	48.55 × 10^15^
Side model	0.25 × 10^−49^	0.50 × 10^−52^
Full model	11.02 × 10^7^	96.84 × 10^14^

*Note.* Scenario 1: large individual differences. Scenario 2: no individual differences. The posterior probability of the null model is the same in every scenario and equals 0.86 × 10^−51^ .

The table shows that the digit model without individual digit effects from scenario 2 has the highest posterior model probability. This is in line with our findings for the normal model. Finally, we can compare the normal model with the highest marginal likelihood to the log-normal with the highest marginal likelihood, that is, the normal digit model from scenario 3 to the log-normal digit model from scenario 2. The log marginal likelihood for the normal digit model without individual differences was 6441.04, while the log marginal likelihood for the log-normal digit model without individual differences was 10,637.45. This indicates that the Bayes factor strongly favors the log-normal digit model over the normal digit model.

The examples illustrate the benefits of comparing hierarchical models of varying complexity. While individual differences are a common phenomenon in psychology, there are instances where general effects are sufficient to adequately describe the data, or where only a random intercept but no random slopes are necessary. As shown in Tables 3 and 4, Bayesian model comparisons can help researchers to identify the model that best describes the data.

## Discussion

In this tutorial, we described model and prior specification, estimation, and model comparison for hierarchical models in the Bayesian framework. In the following, we want to present several key recommendations.

As prior distributions influence model estimation and model comparison, it is important to choose suitable priors. We believe that simulating data based on the chosen priors is an intuitive tool for finding reasonable prior settings that take the existing knowledge in the specific model application context into account. We showed that it can be problematic to use default priors without checking their suitability. Therefore, we recommend performing prior predictive checks with the priors that will be applied before running the analysis.

We illustrated different methods for obtaining Bayes factors. We want to emphasize the importance of checking the variability of the Bayes factor estimate and the Bayes factor sensitivity to prior distributions, and ensuring that the Bayes factor estimate is based on a sufficient number of posterior samples.

This tutorial showed the application of two R packages: *rstan* and *brms*. Which package to use depends mostly on the complexity of the model and your technical background. For the non-technical user, we recommend using *brms* because of its intuitive model specification. However, the default prior setup can be unsuitable for a specific research context and (virtually) always needs to be adapted. Additionally, users are limited to predefined parametrizations. *rstan* offers the greatest flexibility in model and prior specification. This flexibility comes with a cost: if the prior or the model is not well defined, results can be misleading.

### Further recommendations

With this tutorial, we attempted to cover the most relevant topics that researchers encounter when starting with Bayesian hierarchical modeling. However, there are still many remaining issues and recommendations that we could not discuss. Here, we would like to point to further references in the literature if researchers want to continue mastering Bayesian hierarchical modeling.

First, we kept the discussion of the software packages and code fairly brief. We provide further code examples for the digit classification task in the Online Supplement. For different models, it may be useful to study additional tutorials and books. For *rstan*, we recommend the Stan manual (Stan Development Team, 2018a, 2018b). For *brms* there are several useful tutorials (Bürkner, 2017, 2018; Bürkner & Vuorre, 2019). There are also several resources for Bayesian estimation with other samplers (Lee & Wagenmakers, 2013; Rouder et al., 2013). Two other useful tutorials for Bayesian modeling are Schad et al. (2021) and Schad et al. (2022). There are also other R software packages for Bayesian multilevel modeling besides* brms* and *rstan*, such as *BayesFactor* (Morey & Rouder, 2018) and *rstanarm* (Goodrich et al., 2020), that we did not illustrate in this tutorial. However, most of these other R software packages are more limited, for instance, in model specification and prior settings.

Second, we did not discuss study planning. When planning an experiment, researchers may want to determine the number of participants required to gain conclusive results. Bayesian analysis has the advantage that an optional stopping paradigm may be employed (Rouder, 2014; Schönbrodt et al., 2017). Schönbrodt and Wagenmakers et al. (2018) and Stefan et al. (2019) introduced Bayes factor design analysis for fixed and sequential designs. However, these approaches to study planning have not yet been fully extended to a hierarchical setup (but see recent efforts by Vasishth et al., 2023). The extension to nested designs is not entirely trivial because, in addition to planning the number of participants, we also need to consider the number of trials (Rouder & Haaf, 2018). While we think there remain some open questions on the topic, the tutorial by Stefan et al. (2019) is a good place to start for applied researchers.

### Technical possibilities are endless

One key advantage of Bayesian over frequentist hierarchical modeling is that complex modeling leads to fewer convergence issues in the Bayesian framework. This advantage, however, does not imply that there are no technical issues in the Bayesian framework. One possible technical issue is with the choice of priors when using Stan for estimation. The Stan algorithm is most efficient with specific prior distributions. For example, it may be beneficial to use a truncated *t*-distribution on standard deviation parameters instead of an inverse gamma distribution on variances (Gelman, 2006). Using priors not recommended by the Stan team may lead to more issues with convergence. In this tutorial, we highlight that choosing priors based on their predictions on data is beneficial. We still think this is the best path for substantive researchers. If one wants more flexibility in choices of priors or has continuous issues with convergence with their chosen prior distributions, then it might be worth considering using JAGS instead of Stan. JAGS (Plummer, 2003) has a similar functionality and syntax as Stan. While Stan tends to be more efficient in the sampling phase, especially in the case of correlated parameters (Hecht et al., 2021), the JAGS algorithm could be more flexible and less affected by the choice of priors.

Another technical issue may arise with the estimation of Bayes factors. Here, we highlighted two approaches, the Savage–Dickey density ratio (Wagenmakers et al., 2010) and bridge sampling (Gronau et al., 2017). We have already highlighted the drawbacks of the SD density ratio. Bayes factor estimates can also be biased (i.e., display a consistent deviation from the true Bayes factor value). However, bridge sampling has been shown to be relatively unbiased (Sarafoglou et al., 2021). Yet, bridge sampling may also fail in some cases. Failures of the bridge sampling algorithm are identified by unstable Bayes factors across repeated sampling runs. There are improvements to the algorithm that are specifically developed for hierarchical models (Gronau et al., 2019). Nevertheless, assessing the stability of Bayes factor estimates remains crucial.

### Living with uncertainty

Let us reconsider our scenario from the introduction. The graduate student may have read our tutorial and gone back to their supervisor to propose a Bayesian hierarchical analysis of their data. Compared to the initially considered ANOVA, there are many small decisions to be made for this more extensive analysis. These small decisions tend to make researchers uncomfortable. What prior is the right one? Are there enough posterior samples?

In Bayesian modeling, we have to learn to live with these uncertainties. In fact, we recommend embracing them as these small decisions. First, we at the very least are making these decisions deliberately. Second, we can check their impact on the results. Throughout the tutorial we highlighted several potential robustness checks, from prior predictions over convergence assessment, to sensitivity analysis for Bayes factors. These checks may help researchers to understand how robust their results are and when they break. Therefore, we hope the checks will support switching from simple procedures to Bayesian hierarchical modeling. Because, ultimately, the results from such an analysis are much richer.

In summary, by offering a comparison of software packages, guidance on prior selection and default priors, and assessment of the Bayes factor as model comparison method, we hope that Bayesian hierarchical models will become available to a wider psychology public.

## Data Availability

We do not have ownership of the data used in this study, but the data set is publicly available on GitHub (https://github.com/PerceptionCognitionLab/data0/blob/master/lexDec-dist5/). The *.stan* models, results of the model fit, and model comparison are available on our GitHub repository: https://github.com/MyrtheV/Bayesian-Hierarchical-Modelling-An-Introduction-and-Reassessment.

## Footnotes

1 Online Supplement A provides the parameterization for models of aggregated and individual data, to illustrate the difference with hierarchical models.

2 Conjugate priors result in posterior distributions that belong to the same distribution family as the prior distribution (Fink, 1997a). For a normal model, these are the normal distribution for the mean and the inverse gamma distribution for the variance.

3 However, in *rstan*, non-conjugate priors are commonly applied (e.g., Gelman, 2006).

4 Because response times cannot be negative, the prior for the overall baseline *μ*_*γ*_ is a truncated normal with a lower bound of zero.

5 For the overall baseline *μ*_*γ*_ we adjusted the prior based on our expectation of the expected overall response times. A mean parameter of −0.5 for the log-normal distribution corresponds to a mean response time of 1.

6 In our example of the digit classification task we are applying a truncated normal prior distribution for only one parameter. This requires a slightly different *brms* formula and prior specification, explained in the Online Supplement.

7 The design and code for this figure are by van Langen (2020).

8 Note that it would be possible to calculate the posterior model probabilities directly from the marginal likelihoods. However, marginal likelihoods are often not computed explicitly in the model fitting process (e.g., when using the* bayes_factor* function in the *brms* package), so we believe that readers might find it easier to compute posterior model probabilities from Bayes factors.
